# Cost-Effectiveness of Coal Workers' Pneumoconiosis Prevention Based on Its Predicted Incidence within the Datong Coal Mine Group in China

**DOI:** 10.1371/journal.pone.0130958

**Published:** 2015-06-22

**Authors:** Fuhai Shen, Hongbo Liu, Juxiang Yuan, Bing Han, Kai Cui, Yu Ding, Xueyun Fan, Hong Cao, Sanqiao Yao, Xia Suo, Zhiqian Sun, Xiang Yun, Zhengbing Hua, Jie Chen

**Affiliations:** 1 School of Public Health, China Medical University, Shenyang, Liaoning, 110013, P.R. China; 2 School of Public Health, Hebei United University, Tangshan, Hebei, 063000, P.R. China; 3 Occupational Disease Prevention and Treatment Hospital of Datong Coal Mine Group, Datong, Shanxi, 037003, P.R. China; Sun Yat-sen University, CHINA

## Abstract

We aimed to estimate the economic losses currently caused by coal workers’ pneumoconiosis (CWP) and, on the basis of these measurements, confirm the economic benefit of preventive measures. Our cohort study included 1,847 patients with CWP and 43,742 coal workers without CWP who were registered in the employment records of the Datong Coal Mine Group. We calculated the cumulative incidence rate of pneumoconiosis using the life-table method. We used the dose-response relationship between cumulative incidence density and cumulative dust exposure to predict the future trend in the incidence of CWP. We calculate the economic loss caused by CWP and economic effectiveness of CWP prevention by a step-wise model. The cumulative incidence rates of CWP in the tunneling, mining, combining, and helping cohorts were 58.7%, 28.1%, 21.7%, and 4.0%, respectively. The cumulative incidence rates increased gradually with increasing cumulative dust exposure (CDE). We predicted 4,300 new CWP cases, assuming the dust concentrations remained at the levels of 2011. If advanced dustproof equipment was adopted, 537 fewer people would be diagnosed with CWP. In all, losses of 1.207 billion Renminbi (RMB, official currency of China) would be prevented and 4,698.8 healthy life years would be gained. Investments in advanced dustproof equipment would be total 843 million RMB, according to our study; the ratio of investment to restored economic losses was 1:1.43. Controlling workplace dust concentrations is critical to reduce the onset of pneumoconiosis and to achieve economic benefits.

## Introduction

Coal workers' pneumoconiosis (CWP) is the most common occupational disease among coal workers [1–6]. These workers develop CWP because they are exposed to coal dust, mixed dust, and silica dust during the coal mining process. Currently, CWP is the most prevalent occupational disease in the world, especially in developing countries [7–10]. In China, by the end of 2013, there were 750,300 cases of pneumoconiosis [11] and approximately half of them were CWP.

CWP is an incurable and progressive disease. As the disease progresses, patients with CWP gradually lose their livelihood and healthy life years [12]. As a result, patients with pneumoconiosis confer a significant economic burden on families, businesses, and society [13–15]. Great economic benefits could be achieved with dustproof efforts and the resulting reduction in the incidence of pneumoconiosis. One study showed that if dust control devices were installed in all agate-grinding facilities in Khambhat, India, the prevalence of silicosis and tuberculosis would be reduced and financial benefits would be achieved over time [16]. Similarly, the economic losses caused by pneumoconiosis could be avoided by taking effective measures to reduce the incidence of CWP.

Some measures have been taken to prevent or delay the onset of CWP, including technological innovation, wet operation, dust removal by ventilation, individual protection, occupational health management, health education, supervision, and inspection. However, any preventive measure requires a significant up-front monetary investment, while economic losses caused by CWP occur over a long period. As such, future losses are easy to ignore in order to avoid a current investment. Still, all interested parties, including management personnel, laborers, government agencies, health professionals, and advocacy organizations, should know the costs of hazardous working conditions and how to mitigate the risks. Therefore, it is necessary to calculate economic losses currently caused by CWP and, on the basis of these measurements, confirm the economic benefits of preventive measures.

A comprehensive benefit assessment includes a cost-effectiveness analysis and a cost-benefit analysis. The cost-effectiveness analysis refers to a comparative analysis between investment and health effect, which can be expressed in monetary terms [17]. Oxenburgh believed that the cost-benefit model was a relatively simple assessment tool in occupational health economic evaluations. This tool provides important information for decision-makers, since it defines the minimum investment of occupational health resources needed to achieve maximum results and effectiveness [18].

The current revision of “Ordinance of industrial injury insurance” substantially increased compensation to pneumoconiosis patients, which, in effect, increased economic losses attributed to pneumoconiosis [19]. Economic losses can be categorized as direct and indirect. Direct losses include payments for hospital stays, physician visits, allied health services, rehabilitation services, nursing home care, home health care, medical equipment, and insurance administration, as well as payments made directly to patients. Indirect losses include productivity losses, such as wage losses, household productivity losses, and employer productivity losses [20]. Disability-adjusted life years (DALY) is an accepted method for calculating social productivity loss and, for this study, we used DALY to calculate the loss of healthy life and the loss of social productivity [21,22]. We considered an investment in dustproof equipment to include dustproof facilities and supply costs, personal protective equipment costs, occupational health protection training costs, and dust detection and evaluation costs [13,23].

The Datong Coal Mine Group, whose predecessor was the Datong Mining Bureau, was established in 1949 and restructured in July 2000. The Datong Coal Mine Group has experience in artificial exploitation, blasting, and caving processes. After 1970, mechanical mining was widely adopted among the coal mining industry. At the same time, dustproof measures, wet operation, and mechanical ventilation were put into practice in the coal mining process. After 1980, an automated machinery mining process was added. Dust control measures have been gradually strengthened in coal mining workplaces over the last three decades [24]. The Datong Coal Mine Group is representative of the coal mining industry in China for its experience in coal production and dustproof efforts.

For our study, we evaluated dust-exposed workers with and without CWP from the Datong Coal Mine Group. The goals of our study were to analyze the prevalence of CWP and predict the number of CWP cases among workers who first experienced dust exposure between 1970 and 2010; to calculate economic losses and healthy life losses due to CWP; and to calculate economic and social benefits achieved as a result of dustproof investments. This is important information for occupational health management departments and coal mining enterprises. We aimed to provide a theoretical basis for the formulation and revision of laws, regulations, and standards for the prevention of pneumoconiosis in China.

## Study Population and Methods

### Study population

Our study cohort were conducted in 2012 in Datong Coal Mine Group at a total of 15 mines. Coal workers were included in our study if their dust exposure started between January 1, 1970 and December 31, 2010 as part of the Datong Coal Mine Group and if they were exposed to dust for at least one year. We required physical examination cards, detailed records of occupational history, and posterior-anterior chest radiographs for all workers enrolled in our study. The study was approved by the Medical Ethics Committee of China Medical University (permit number CMU6206-3008). The need for written consent was not deemed necessary and was waived by the Medical Ethics Committee. The study was carried out in accordance with the approved guidelines. In order to protect the privacy of research subjects and the confidentiality of their personal information, Identifying information, including names, initials, identity card numbers, or job serial numbers of the subjects, did not exsit in our written descriptions.

We constructed a database, which included demographic details, work history records including the dates of dust exposure, individual medical records, pneumoconiosis diagnosis records, dust concentrations of the workplace, economic losses attributed to pneumoconiosis patients, and costs associated with dustproof facilities. The demographic details and work history records were obtained from personnel files in the human resources department. The individual medical and pneumoconiosis diagnosis records were obtained from the occupational disease prevention and treatment hospital. The dust concentrations of the workplaces were obtained from the department of dust detection and monitoring. The economic loss data were obtained from department of industrial injury insurance, hospital, and finance. The costs of dustproof equipment and supplies were obtained from the equipment department and finance department.

### Diagnosis of pneumoconiosis

The diagnosis of CWP was based on the “Diagnostic criteria of pneumoconiosis” and corresponding standard films of pneumoconiosis in China [25]. The Chinese “Diagnostic criteria of pneumoconiosis” was formulated based on the “Guidelines for the use of the ILO international classification of radiographs of pneumoconioses” [26]. They are based on the same principle. Pneumoconioses were classified as stage Ⅰ, Ⅱ, and Ⅲ in Chinese Diagnostic criteria of pneumoconiosis based on the ILO criteria. Chest radiographs of subjects were read and the diagnosis was made independently by five qualified experts who were members of the pneumoconiosis diagnosis committee. If the diagnoses differed among the five experts, the opinion of the majority ruled. The experts identified disabilities associated with pneumoconiosis based on the "Standard of work-related disability identification" (Table A in S1 File) [27].

### Occupational categories

We defined four work areas in the underground mines of the Datong Coal Mine Group by reviewing the work history of the subjects [28,29]: tunneling, mining, combining, and helping. Workers who consistently worked in the same area were defined by their respective work area titles: tunneling, mining, combining, or helping. The duration of dust exposure for each coal worker was the sum of years of each dust exposure job. The duration of each dust exposure job was measured from the start date to the end date of the job. Workers were classified as “tunneling” if their duration in tunneling areas accounted for more than half of the entire duration of dust exposure. Workers were classified as “mining” if they worked in tunneling areas for less than 2 years and their duration in mining areas accounted for more than half of the entire duration of dust exposure. Workers were classified as “combining” if their duration in tunneling areas was more than 2 years but less than half of the entire duration of dust exposure. Workers were classified as “helping” if they could not be included in the tunneling, mining, or combining categories.

### Dust exposure data

Dust was collected on a membrane filter and measured by gravimetric method, which is the national standard of measurement for dust concentration [30,31]. This information was then used to calculate the cumulative dust exposure (CDE) for each coal worker [32]. CDE is usually estimated according to the following formula [8] (Eq 1):

$$\text{CDE}=\sum_{j=1}^{n}\left(Cj\times Tj\right)$$ Eq 1

In the formula, CDE is calculated in milligrams/cubic meter-years (mg·years); n is the total number of job titles held by the individual during his work history; Cj is the 8-hour time-weighted mean concentration of dust in milligrams/cubic meter for the *j*th job title within a facility and an employment period; and Tj is the duration of employment in years in the *j*th job.

### Calculation of the cumulative incidence rate

We established four subcohorts according to occupational categories: tunneling, mining, combining, and helping. We calculated the cumulative incidence rate of CWP in the corresponding years of observation for each cohort using the life-table method [23,28]. The rates were analyzed by Peto’s log-rank test. We calculated the cumulative incidence rates, using the tunneling cohort as an example in Table B in S1 File. Cumulative incidence rates of the other cohorts were calculated in the same manner.

### Predicted future incidence of CWP

We calculated the dose-response relationship between cumulative incidence density (CID) and CDE for the four occupational categories. We also introduced the variable of person-years in order to account for time in the equation. The incidence density of each dose group was the ratio of incidence number and the adjusted observed person-years. CID was calculated by the life-table method. We translated CDE into its natural logarithm and translated CID into its logit value, according to the following formula (Eq 2):

$$\text{logit}=\ln\left(\frac{P}{1-P}\right)$$ Eq 2

By this method, we obtained the linear regression equation for the logit and the natural logarithm of CDE. We calculated the estimated values of incidence density from the equation that was used to predict the future incidence of CWP at the dust concentration level of 2011 and after adopting dust-reduction measures.

### Compensation standards of direct economic loss caused by CWP

Direct economic losses included medical costs, lump-sum grants for disability, disability allowances, lump-sum grants for death, funeral grants, nursing costs, food allowances, traffic fees, and pension costs for dependent relatives (Table C in S1 File) [14,33,34]. The total amount of compensation was calculated according to the 2010 revision of “Ordinance of industrial injury insurance” [19].

Medical costs included inspection fees, hospitalization fees, and medicine and treatment-related costs. The total medical cost for each CWP patient was calculated. Each CWP patient was compensated with a lump-sum grant for disability (Table D in S1 File). Disability allowances were only distributed to patients whose disability grades ranged from 2 to 6 before retirement (Table E in S1 File). Funeral grants equals the average monthly wage of the staff and workers in a region multiplied by 6 months, which were distributed to relatives of deceased CWP patients. If the disabled workers died during the period of suspension with pay caused by CWP, their relatives were compensated with a lump-sum grant for death. Nursing costs were compensated to CWP patients who required nursing care from industrial injury insurance funds (Table F in S1 File). Food allowances were paid to those who required medical treatment, which is 35 RMB per day in Shanxi Province. Dependent relative pension costs were paid to the families of deceased CWP patients whose disability grades ranged from 2 to 4 or CWP patients who died during the period of suspension with pay regardless of disability grade, if the relatives relied on CWP patients when they were alive. Traffic fees were compensated to CWP patients who traveled to other areas for medical treatment. In our study, very few CWP patients traveled to other areas for medical treatment, so we did not calculate this factor into the total economic loss.

### Compensation standards of indirect economic loss caused by CWP

Indirect economic losses included social productivity losses caused by CWP patients, costs of training new employees, traffic fees for people who accompanied CWP patients, social productivity losses caused by people who accompanied CWP patients, and losses owed to stopping or cutting production (Table C in S1 File) [15].

Social productivity losses attributed to pneumoconiosis patients were calculated using ∑DALY, gross domestic product (GDP) per capita, productivity weight [22]. DALY was used to evaluate life loss caused by pneumoconiosis, which includes years lived with disability (YLD) and years of life lost (YLL) [21,22]. If coal mine workers suffered from CWP before retirement, new workers were recruited to supplement their jobs; the cost of training new supplemental employees was included in the economic losses caused by pneumoconiosis. Traffic fees for people who accompanied hospitalized CWP patients were also included in the economic losses caused by pneumoconiosis. According to travel standards of China's residents, the average annual per capita traffic fee was 2,000 RMB. Traffic fees for accompanying people were the average daily traffic costs multiplied by the number of days spent in the hospital. Social productivity losses caused by accompanying people were included in the economic losses caused by pneumoconiosis; it was calculated as the product of GDP, productivity weight, and length of disability of pneumoconiosis patients. Since coal mining facilities did not stop or limit production because of dust hazards, there was no economic loss related to stopping or cutting production.

### Economic effectiveness of investment in dustproof equipment and practices

We calculated the economic benefits of dustproof to be the difference between the monetary investment in dustproof equipment and practices and the restored economic losses owed to the investment. We used current CWP patients as the example for calculating economic losses and we used prices and costs in 2011 as the standards for compensation and costs. Further, social benefits were calculated by assessing the restoration of healthy life years of the workers exposed to dust.

## Results

### Baseline characteristics

There were 49210 people in Datong Coal Mine Group who started to expose to dust from 1970 to 2010. 3621 people were excluded because of incomplete data, such as any drop outs, workers loss to follow up, or workers who die from other disease. 45589 subjects met the requirements, the coincidence rate was 92.6%. We evaluated a total of 45,589 coal workers in our study, including 1,847 patients with CWP and 43,742 workers without CWP. The average age of CWP onset for the 1,847 CWP patients was 45.9 ± 8.3 years and the average duration of dust exposure for these patients was 19.8 ± 7.2 years. In 2011, the average age of the 43,742 coal workers without CWP was 42.3 ± 9.6 years and the average duration of dust exposure for these workers was 18.9 ± 9.5 years. We observed statistically significant differences between the dust-exposed workers with CWP and without CWP in years of first dust exposure, occupational category, and CDE, according to the chi square test (Table 1).

**Table 1:** Characteristics of coal workers with and without coal workers’ pneumoconiosis (CWP).

Characteristic		With CWP (n = 1847)(%)	Without CWP (n = 43742)(%)	χ^2^	P
Years of first dust exposure	1970s	1,217(65.9)	5,848(13.4)	3.7×10^3^	<0.001
	1980s	630(34.1)	3,7894(86.6)		
Occupational category	Tunneling	735(39.8)	2,634(6.0)	3.6×10^3^	<0.001
	Mining	619(33.5)	1,0160(23.2)		
	Combining	297(16.1)	4,721(10.8)		
	Helping	196(10.6)	26,227(60.0)		
Cumulative dust exposure	<100 mg·years	168(9.1)	26,589(60.8)	4.5×10^3^	<0.001
	≥ 100 mg·years	218(11.8)	9,828(22.5)		
	≥ 2000 mg·years	1,461(79.1)	7,325(16.7)		

**Fig 1:**
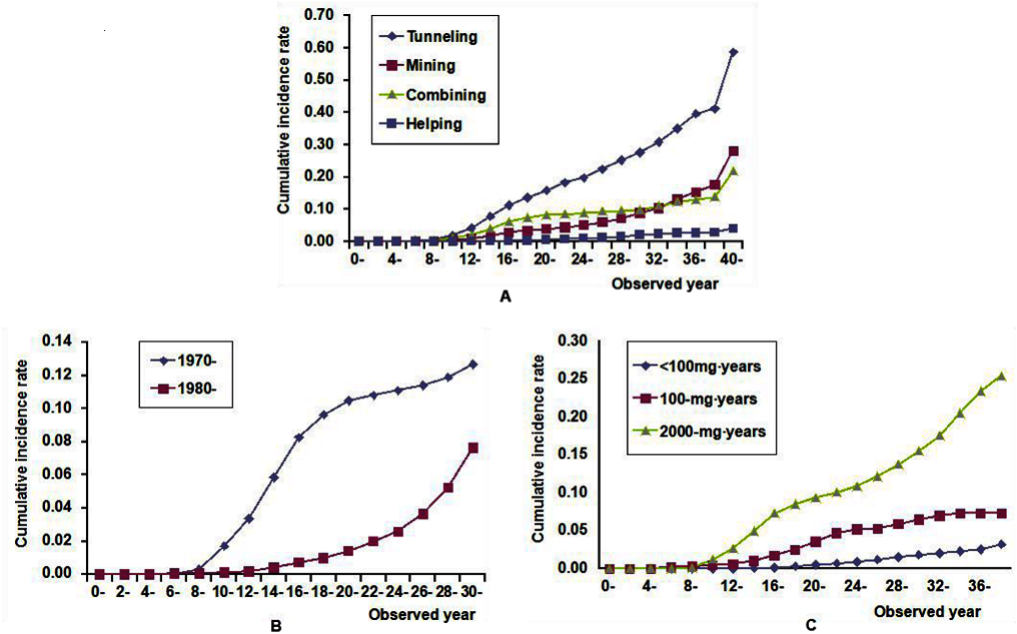
Cumulative incidence rate of CWP among coal workers. (A) Cumulative incidence rate of CWP in different occupational categories: tunneling cohort vs mining cohort (χ^2^ = 597.7; P < 0.001), tunneling cohort vs combining cohort (χ^2^ = 228.9; P < 0.001), tunneling cohort vs helping cohort (χ^2^ = 3,116.7; P < 0.001), mining cohort vs combining cohort (χ^2^ = 17.3; P < 0.001), mining cohort vs helping cohort (χ^2^ = 654.4; P < 0.001), and combining cohort vs helping cohort (χ^2^ = 827.9; P < 0.001). (B) Cumulative incidence rate of CWP according to years of first dust exposure: 1970s vs 1980s (χ^2^ = 27,432.5; P < 0.001). (C) Cumulative incidence rate of CWP according to cumulative dust exposure: ≥ 2000 mg·years group vs ≥ 100 mg·years group (χ^2^ = 338.1; P < 0.001), ≥ 2000 mg·years group vs < 100 mg·years group (χ^2^ = 1,867.9; P < 0.001), and ≥ 100 mg·years group vs < 100 mg·years group (χ^2^ = 308.9; P < 0.001).

The cumulative incidence rates of CWP of tunneling, mining, combining, and helping cohorts were 58.7%, 28.1%, 21.7%, and 4.0%, respectively, during the 42-year observation period (Fig 1A). We observed significant differences between occupational categories, according to the log-rank test. The cumulative incidence rate of CWP in the group of workers first exposed to dust in the 1970s was 12.7% and the rate in the group first exposed to dust in the 1980s was 7.6% during the 32-year observation period (Fig 1B). The cumulative incidence rates were 25.4%, 7.3%, and 3.2% among coal workers whose CDEs were ≥ 2000 mg·years, ≥ 100 mg·years, and <100 mg·years during the 42-year observation period (Fig 1C). The dust concentration decreased with time in different work areas (Table 2). We observed a dose-response relationship between cumulative incidence rate and CDE (S1 Fig).

**Table 2:** Geometric means (+1 SD, -1 SD) of dust concentration (mg/m^3^) in the working areas according to years.

Years	Tunneling area		Mining area		Combining area		Helping area	
	Geometric mean	Range	Geometric mean	Range	Geometric mean	Range	Geometric mean	Range
1970s	168.2	(242.1, 116.8)	144.1	(272.9, 76.1)	142.3	(279.8, 72.3)	4.9	(6.4, 3.8)
1980s	156.4	(351.8, 69.5)	145.2	(248.8, 84.7)	120.3	(243.3, 59.5)	4.4	(5.8, 3.3)
1990s	123.4	(376.2, 40.5)	77.9	(136.7, 44.4)	68.6	(137.1, 34.4)	1.5	(3.4, 0.7)
2000s	78.2	(182.5, 33.5)	52.5	(72.2, 38.1)	42.8	(77.3, 23.7)	0.3	(0.4, 0.2)

### Prediction model of CWP

**Fig 2:**
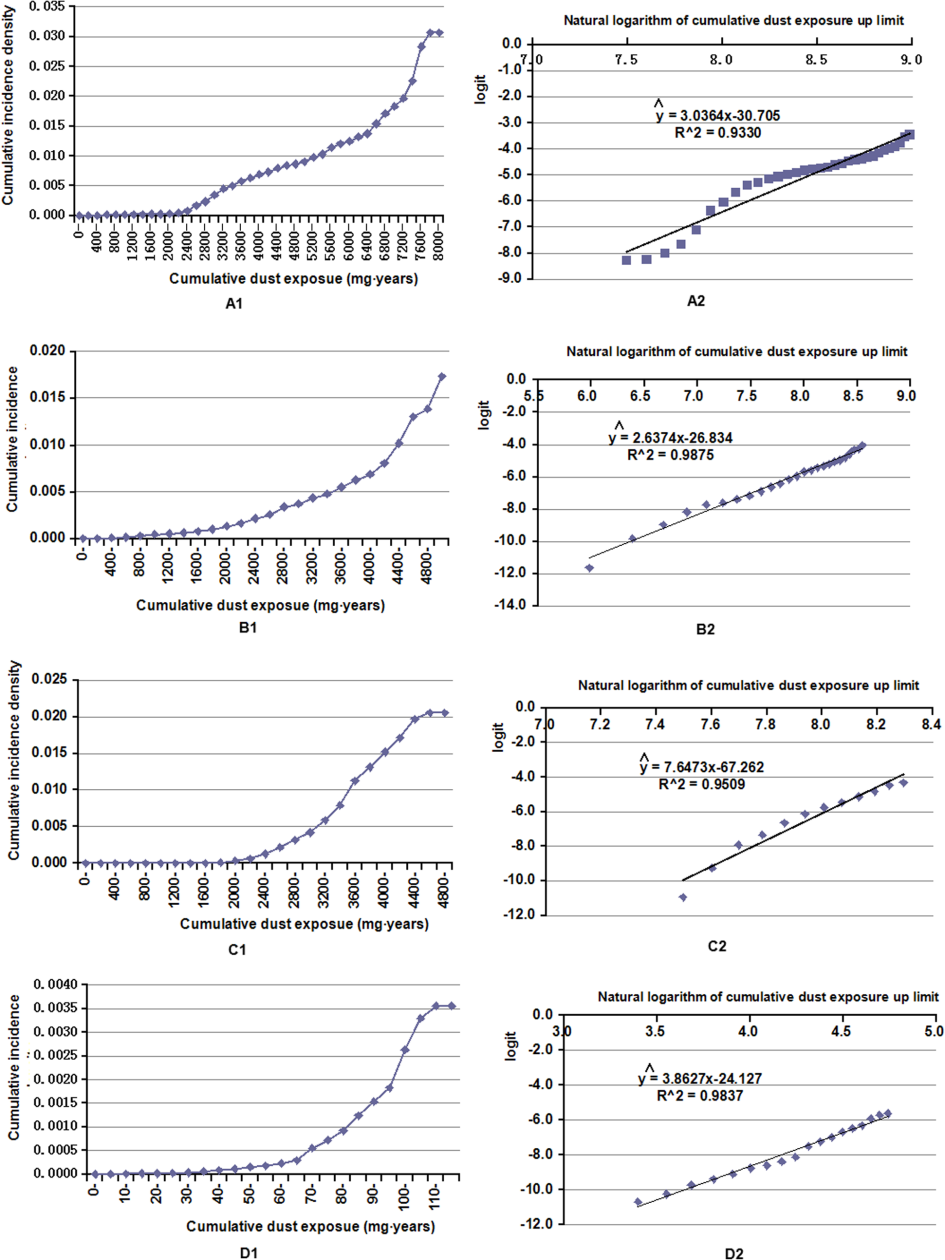
Dose-response relationship between cumulative incidence density and cumulative dust exposure in the cohorts and their variable transformation. (A1) Dose-response relationship between cumulative incidence density and cumulative dust exposure in the tunneling cohort. (A2) Dose-response relationship between the logit and the natural logarithm of cumulative dust exposure in the tunneling cohort. (B1) Dose-response relationship between cumulative incidence density and cumulative dust exposure in the mining cohort. (B2) Dose-response relationship between the logit and the natural logarithm of cumulative dust exposure in the mining cohort. (C1) Dose-response relationship between cumulative incidence density and cumulative dust exposure in the combining cohort. (C2) Dose-response relationship between the logit and the natural logarithm of cumulative dust exposure in the combining cohort. (D1) Dose-response relationship between cumulative incidence density and cumulative dust exposure in the helping cohort. (D2) Dose response relationship between the logit and the natural logarithm of cumulative dust exposure in the helping cohort.

The cumulative incidence densities were calculated for each group of cumulative dust exposure by using method of life table for tunneling workers (Table G in S1 File). We observed a dose-response relationship between CDE and cumulative incidence density estimation (Fig 2A1). After logarithmic transformation of variables, the following linear equation of the two variables was obtained (Eq 3) (Fig 2A2):

logit=3.0364×ln(CDE)-30.705. Eq 3

In the same way, we observed a dose-response relationship between CID and CDE for mining workers (Fig 2B1). The linear equation for the mining workers was (Eq 4) (Fig 2B2):

logit=2.6374×ln(CDE)-26.834. Eq 4

The dose-response relationship for combining workers is shown in Fig 2C1. The linear equation of the combining workers was (Eq 5) (Fig 2C2):

logit=7.6473×ln(CDE)-67.262. Eq 5

The dose-response relationship for helping workers is shown in Fig 2D1. The linear equation of the mining workers was (Eq 6) (Fig 2D2):

logit=3.8627×ln(CDE)-24.127. Eq 6

The incidence density estimation for each segment was calculated by these equations. The tunneling cohort is used as an example in Table H in S1 File.

### Prediction of future CWP cases assuming the dust levels of 2011

For our prediction of new cases of CWP, we assumed that future dust concentrations would remain at the level of those in 2011, which were 31.6 mg/m^3^, 23.2 mg/m^3^, 23.2mg/m^3^, and 0.2 mg/m^3^ in the tunneling, mining, combining, and helping areas, respectively. According to the incidence density estimation of tunneling, mining, combining, and helping cohorts, we predicted the number of future CWP cases within the life expectancy of each subject to be 1,422.1, 1,254.9, 1,195.8, and 427.3 among tunneling (Tables I & J in S1 File), mining (Table K in S1 File), combining (Table L in S1 File), and helping cohorts (Table M in S1 File), respectively. We predicted a total of 4300 new CWP cases among workers in the four occupational categories who had their first dust exposure between 1970 and 2010, if workplace dust concentrations remain at the levels of those in 2011 (Table 3).

**Table 3:** Prediction of future CWP cases at the dust levels of 2011 and after implementation of dustproof equipment and practices.

Occupational category	n	Predicted number of cases at the dust levels of 2011	Predicted number of cases after dustproof	Difference
Tunneling	2,634	1,422.1	1,245.5	176.6
Mining	10,160	1,254.9	1,084.7	170.2
Combining	4,721	1,195.8	1,020.5	175.3
Helping	26,227	427.3	412.5	14.8
Total	43,742	4,300.0	3,763.2	536.9

### Prediction of future CWP cases after decreasing dust levels

Dustproof equipment could decrease workplace dust concentrations by eighty percent of the present levels [35]. To assess the potential decline in CWP after dustproof, we calculated the CDE for each coal worker until his retirement assuming a dust concentration at 20% of the 2011 level. The assumed dust concentrations were 6.3 mg/m^3^, 4.6 mg/m^3^, 4.6 mg/m^3^, and 0.04 mg/m^3^ in the tunneling, mining, combining, and helping areas, respectively. According to the incidence density estimations of the tunneling, mining, combining, and helping cohorts, we predicted the number of future CWP cases within the life expectancy of each subject to be 1,245.5, 1,084.7, 1,020.5, and 412.5 among tunneling (Table N in S1 File), mining (Table O in S1 File), combining (Table P in S1 File), and helping cohorts (Table Q in S1 File), respectively. We predicted that 3,763 coal workers would develop CWP after the implementation of advanced dustproof equipment and practices, which equates to 537 fewer people with CWP (Table 3).

### Direct economic losses attributed to CWP patients

According to our investigation, the average annual medical cost of each pneumoconiosis patient was 23,137.5 RMB. The average course of pneumoconiosis disease was 31.6 years, and the average total expense of each case was 730,394.1 RMB. The total medical cost of the 1,847 pneumoconiosis patients over the entire course of the disease was 1,349,037,953.3 RMB (Table R in S1 File).

The average monthly salary of underground coal mine workers in the Datong Coal Mine Group was 8,056.2 RMB. The total lump-sum disability grant was calculated to be 248,717,519.0 RMB (Table S in S1 File).

In this study, 1,047 CWP patients had disability grades that ranged from 2 to 6 before retirement. The total disability allowance for CWP patients was calculated to be 1,199,356,930.5 RMB, based on the costs in 2011 (Table T in S1 File).

The average monthly wage in Shanxi Province was 3,269.2 RMB in 2011 [36]. Funeral grants for the 1,847 CWP patients were calculated to be 36,228,941.9 RMB (3,269.2 RMB × 6 months × 1847 persons), based on the costs in 2011. Funeral grants for persons who did not die from work are 2,000 RMB [37]. Therefore, in our study, funeral grants totaled 32,534,941.9 RMB [36,228,941.9 RMB–(2,000 RMB × 1,847 persons)].

Of the 1,847 patients with CWP, 23 died during the period of suspension with pay. The average wage of workers in China was 41,799 RMB in 2010 [36]. For our investigation, we calculated the lump-sum grants for death to be 19,227,540 RMB (41,799 RMB × 20 times × 23 persons).

The average annual wage of workers in Shanxi Province in 2011 was 39,230 RMB [36]. The total nursing costs were calculated to be 257,314,866.1 RMB(Table U in S1 File).

A daily food allowance of 20 RMB was subsidized to hospitalized CWP patients in Shanxi Province. In 2011, the average length of hospitalization of CWP patients from the Datong Coal Mine Group was 75 days. The average disease duration of the 1,847 CWP patients was 31.6 years. Therefore, we calculated the total food allowance to be 87,464,685 RMB (20 RMB × 75 days × 1,847 persons × 31.6 years).

In 2011, the average dependent relative pension cost for deceased CWP patients from the Datong Coal Mine Group was 17,782.32 RMB. By the end of 2011, the average age of death of the 210 CWP patients who died was 55.4 years. The average life expectancy of the patients was 80.1 years. The average life loss was 24.7 years. Therefore, we calculated the total dependent relative pension costs to be 294,480,198.3 RMB (17,782.32 RMB × 671 persons × 24.7 years) for 655 CWP patients whose disability grade ranged from 2 to 4 and for 16 CWP patients whose disability grade was 7 but died during the period of suspension with pay.

For our analysis, we calculated the direct economic losses caused by 1,847 CWP patients to total 3,488,134,634.1 RMB, assuming prices and costs of 2011. The average direct economic loss was 1,888,540.7 RMB per patient with CWP (Table 4). Medical costs accounted for 38.7% and disability allowances accounted for 34.4% of total direct economic loss.

**Table 4:** Direct economic losses attributed to patients with CWP.

Category	n	Economic loss (RMB)	Proportion of total direct economic loss (%)
Medical costs	1,847	1,349,037,953.3	38.7
Lump-sum grants for disability	1,847	248,717,519.0	7.1
Disability allowances	1,047	1,199,356,930.5	34.4
Funeral grants	1,847	32,534,941.9	0.9
Lump-sum grants for death	23	19,227,540.0	0.6
Nursing costs	655	257,314,866.1	7.4
Food allowances	1,847	87,464,685.0	2.5
Dependent relative pension costs	671	294,480,198.3	8.4
Total	1,847	3,488,134,634.1	100.0

### Indirect economic losses attributed to CWP patients

At the end of our observation period, among 1,847 patients with CWP, 1,637 patients were alive and 210 cases were deceased. Eight patients had a disability grade of 2, 147 had a grade of 3, 500 had a grade of 4, 432 had a grade of 6, and 760 had a grade of 7. The age of CWP onset ranged from 22.0 to 75.9 years, and the average age was 45.9 years (Table V in S1 File). Total YLD was 13,313.4 years for all of the 1,847 patients with CWP (Table W in S1 File). Total YLL was 2,848.8 years for the 210 deceased CWP patients (Table X in S1 File). Total DALY was 16,162.2 years for the 1,847 patients with CWP. The average DALY was 8.8 years for each patient with CWP (Table Y in S1 File).

In 2011, China's per capita GDP was 35,181 RMB [36]. The economic social productivity loss caused by CWP was 426,239,926.3 RMB, according to productivity weights of the different age groups (Table Z in S1 File).

In our study, 1,730 of the 1,847 coal workers with CWP experienced disease onset before retirement. Training each new coal worker cost approximately 1,286 RMB in the Datong Coal Mine Group. Therefore, we calculated the total cost of training new supplemental employees to be 2,224,780 RMB (1,286 RMB × 1,730 persons).

According to travel standards for China's residents, the average annual traffic fee per capita was 2,000 RMB. Based on our investigation, the average duration of pneumoconiosis was 31.6 years and patients were hospitalized for an average of 75 days per year. For this analysis, we assumed that each hospitalized patient required one accompanying family caregiver. Therefore, we calculated traffic fees to be 23,962,927.4 RMB (2,000 RMB × 1,847 persons × 31.6 years × 75 days/365 days/year).

In 2011, China's GDP per capita was 35,181 RMB [36] and the productivity weight of the total population was 0.5. Therefore, we calculated the social productivity loss caused by accompanying family members, assuming one per hospitalized patient, to be 210,759,937.2 RMB (35,181 RMB × 1,847 persons × 0.5 × 31.6 years × 75 days/365 days/year).

The total indirect economic losses caused by CWP patients totaled 663,187,570.9 RMB. The average loss was 359,062.0 RMB per patient (663,187,570.85 RMB / 1,847 persons). Social productivity loss caused by CWP patients accounted for 64.3% and social productivity loss caused by accompanying family members accounted for 31.8% of the total indirect cost (Table 5).

**Table 5:** Indirect economic losses caused by patients with CWP.

Category	Economic loss (RMB)	Proportion of total indirect economic loss (%)
Social productivity loss caused by CWP patients	426,239,926.3	64.3
Cost of training new supplemental employees	2,224,780.0	0.3
Traffic fees for accompanying family members	23,962,927.4	3.6
Social productivity loss by accompanying family members	210,759,937.2	31.8
Total	663,187,570.9	100.0

### Total economic losses caused by CWP patients

On the basis of our calculations, we concluded that the total economic loss caused by 1,847 CWP patients who experienced their first dust exposure between January 1,1970 and December 31, 2010 was 4,151,322,205.0 RMB (3,488,134,634.1 RMB + 663,187,570.9 RMB). The average loss attributed to each patient was 2,247,602.7 RMB (4,151,322,205.0 RMB / 1,847 persons). Direct economic loss accounted for 84.0% and indirect economic loss accounted for 16.0% of total economic loss. Direct economic loss was 5.3 times greater than the indirect economic loss (Table AA in S1 File).

### Cost-effectiveness analysis of future dustproof investments

If the 43,742 coal mine workers in our study continued working from January 1, 2012 until their retirement, the average dust exposure time would be 17.73 years. If new equipment, such as an underground water purification system and a high-pressure spray system with pneumatic power, was adopted, workplace dust concentrations would decrease by 80% of the present levels [35]. According to the results of our study, the average number of working areas (tunneling, mining, and combining) and trans-shipment points were 13 and 37, respectively. Working areas included the fully mechanized coal mining area, general machine mining area, blasting mining area, rock roadway, coal roadway, and half coal-rock roadway in the 15 mines of the Datong Coal Mine Group. New equipment and related maintenance costs of dustproof initiatives would be 6 million RMB in the first year and 3 million RMB in each subsequent year. Assuming that the new equipment was adopted in all 15 mines, the cost would total 843 million RMB for 17.73 years [(6 million RMB × 1 year + 3 million RMB × 16.73 years) × 15]. If the dustproof equipment and practices were adopted, dust concentrations would decrease to 20% of the present levels. As a result, 537 fewer coal workers would develop CWP and 1.207 billion RMB (2.248 million × 537 persons) in economic losses would be restored. Economic benefits of 364 million RMB (1,207 million—843 million) and 4,698.8 healthy life years (8.8 years × 537 persons) would be obtained by implementing dust-reduction strategies.

## Discussion and Conclusions

Occupational injuries and diseases occur as a result of the production of goods and services. In other words, they are economic phenomena [38]. The failure to recognize this central aspect of occupational health limits the effectiveness of interventions ostensibly designed to prevent disease and injury [13]. Currently, pneumoconiosis is the primary occupational disease in China [11] and the government has assigned great importance to the prevention and treatment of pneumoconiosis. In 1987, "Pneumoconiosis disease prevention and control regulations of the People's Republic of China" was promulgated and implemented [39]. Additionally, in 2001, "Law of the People’s Republic of China on Prevention and Control of Occupational Diseases" was promulgated; it was revised in 2011 [40]. The implementation of these laws and regulations provided legal guarantees for the prevention and treatment of pneumoconiosis.

Despite laws aimed at prevention, the cumulative incidence rates of CWP remained high among the Datong Coal Mine Group. We evaluated the number of patients with CWP who experienced their first dust exposure between 1970 and 2010 in the Datong Coal Mine Group. We predicted that, among all these workers, 4,300 would develop CWP in the future if workplace dust concentrations remained at the levels of 2011. We predicted that 3,763 coal workers would develop CWP if advanced dustproof strategies were adopted, which equates to 537 fewer cases of CWP; losses of 1.207 billion RMB would be restored and 364 million RMB and 4,698.8 healthy life years would be gained. Further, dustproof strategies would benefit more than the people who first experienced dust exposure between 1970 and 2010; workers who started work in the mines after 2011 would also benefit. Including these newer workers in the analysis would more than double the 537 patients who would not develop CWP and greater economic effectiveness would be realized.

In our survey, direct economic loss exceeded indirect economic loss and accounted for 84.0% of total economic loss. This result was different from previous research in China [15], but it was similar to reports from other developed countries [20]. Our finding might be the result of increases in lump-sum grants for disability, lump-sum grants for death, and medical costs, which was a promotion for the prevention and treatment of pneumoconiosis.

Dust concentrations in the workplaces of the Datong Coal Mine Group have decreased significantly since the implementation of a series of dustproof measures, but the levels are still not controlled under the standards defined in “Occupational exposure limit (OEL) for hazardous agents” [8,41]. Dust is still the primary occupational hazard in the Datong Coal Mine Group, as well as other coal enterprises. If dust concentrations decrease, the incidence of CWP will decrease.

The implementation of advanced dustproof equipment, such as a high-pressure spray system with pneumatic power and an underground water purification system, would require an investment of 843 million RMB, according to our analysis, but the dust concentrations would subsequently decline in the coal mines. For example, we expect the dust level to be reduced to 6.3 mg/m^3^ in the tunneling area. At this level, if a worker was exposed to dust for 40 years, his CDE would be 252.8 mg·years. According to the results of our study, the risk probability of developing CWP of tunneling workers with a CDE of 200 mg·years was only 0.0064. The majority of workers would, therefore, be free from pneumoconiosis, and 1.207 billion RMB would be restored. We calculated the ratio of dustproof investments and restored economic losses to be 1:1.43 (843/1,207). Controlling workplace dust concentrations is critical to reduce the onset of pneumoconiosis and to achieve economic benefits.

It is important to recognize pneumoconiosis prevention as an economic activity [13]. In our investigation, we observed substantial economic losses and healthy life losses for CWP patients who were first exposed to dust between 1970 and 2010 in the Datong Coal Mine Group. Improving dust protection could bring considerable economic benefits to the coal mining industry owing to a reduced number of CWP cases. Dust also causes a variety of diseases of the respiratory system and it can increase the incidence rate of other chronic diseases [42,43]. If the economic losses caused by other diseases related to dust were considered, including physical and mental pain to patients and their families, broken families, and social instability, the losses would total more than our original estimates. Such estimates would be a more powerful illustration of the importance of dust control and exposure prevention. If funds are invested early in dustproof equipment and strategies, great benefits will be realized owing to delayed effects of pneumoconiosis [44].

In conclusion, implementing dustproof devices and practices would not only reduce the incidence of CWP but also, in the long run, increase financial profits. Occupational health management departments and coal mining enterprises can benefit from an understanding of this information.

## Supporting Information

S1 Fig Dose-response relationship between cumulative incidence rate of CWP and cumulative dust exposure. (TIF)

S1 File Supplementary tables A-AA. **Table A.** The relationship between the disability grade of occupational disease and the stage of pneumoconiosis. **Table B.** Cumulative incidence rate of CWP in the tunneling cohort. **Table C.** Contents included in direct and indirect economic loss caused by CWP. **Table D.** The compensation standard of lump-sum grant of disability for pneumoconiosis. **Table E.** The compensation standard of disability allowance for pneumoconiosis before retirement. **Table F.** The compensation standard of nursing cost for pneumoconiosis. **Table G.** Cumulative incidence density of CWP for tunneling workers. **Table H.** Logit variable transformation and incidence density estimation for tunneling workers. **Table I.** The number of CWP prediction for tunneling workers in the future if dust concentration maintained at the level of 2011. **Table J.** The number of CWP prediction for tunneling workers in the future if dust concentration maintained at the level of 2011. **Table K.** The number of CWP prediction for mining workers in the future if dust concentration maintained at the level of 2011. **Table L.** The number of CWP prediction for combining workers in the future if dust concentration maintained at the level of 2011. **Table M.** The number of CWP prediction for helping workers in the future if dust concentration maintained at the level of 2011. **Table N.** The number of CWP prediction for tunneling workers in the future if advanced dustproof measures was adopted. **Table O.** The number of CWP prediction for mining workers in the future if advanced dustproof measures was adopted. **Table P.** The number of CWP prediction for combining workers in the future if advanced dustproof measures was adopted. **Table Q.** The number of CWP prediction for helping workers in the future if advanced dustproof measures was adopted. **Table R.** Medical costs attributed to CWP. **Table S.** Lump-sum grants for disability caused by CWP. **Table T.** Allowances for disability caused by CWP. **Table U.** Nursing costs attributed to CWP. **Table V.** The age of onset distribution of CWP in each occupational character. **Table W.** Years lived with disability (YLD) by CWP patients. **Table X.** Years of life lost (YLL) by CWP patients. **Table Y.** Disability adjusted life years (DALY) caused by CWP (year). **Table Z.** Economic losses attributed to decreased social productivity by patients with CWP. **Table AA.** Total economic loss caused by CWP (RMB)
(DOC)

S2 File Supplementary raw anonymized dataset. (XLS)
