# Supplementary figures and images for: Cost-Effectiveness of Coal Workers' Pneumoconiosis Prevention Based on Its Predicted Incidence within the Datong Coal Mine Group in China
Source: PLoS One. 2015 Jun 22;10(6):e0130958. doi: 10.1371/journal.pone.0130958 (PMC4476760; doi:10.1371/journal.pone.0130958)

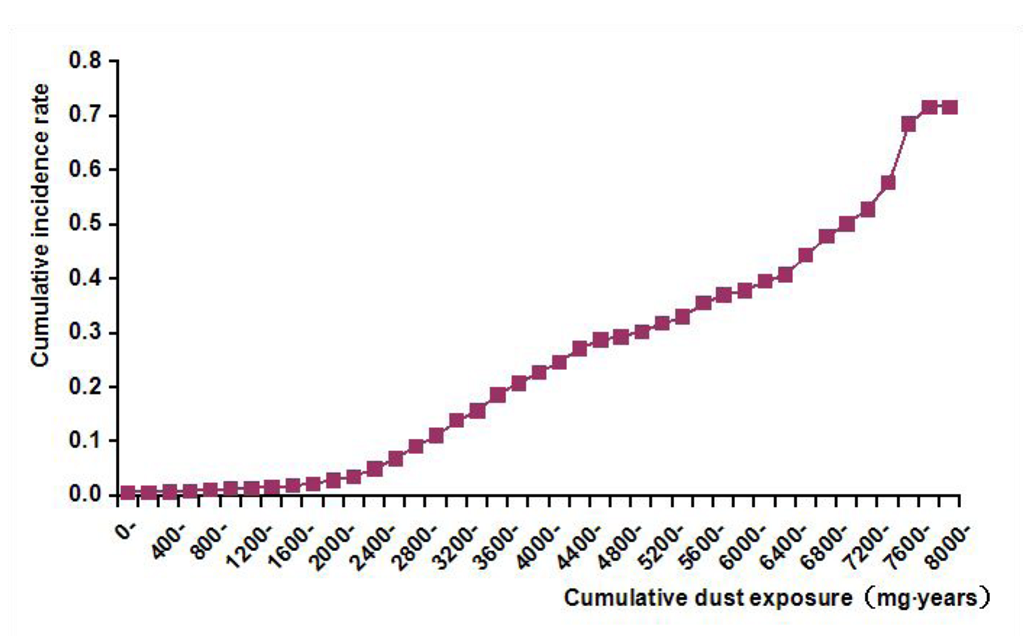

Supplement: S1 Fig — (TIF) [file pone.0130958.s001.tif]
