# Supplementary material for: Cost-Effectiveness of Coal Workers' Pneumoconiosis Prevention Based on Its Predicted Incidence within the Datong Coal Mine Group in China
Source: PLoS One. 2015 Jun 22;10(6):e0130958. doi: 10.1371/journal.pone.0130958 (PMC4476760; doi:10.1371/journal.pone.0130958)
Supplement: S1 File — Table A. The relationship between the disability grade of occupational disease and the stage of pneumoconiosis. Table B. Cumulative incidence rate of CWP in the tunneling cohort. Table C. Contents included in direct and indirect economic loss caused by CWP. Table D. The compensation standard of lump-sum grant of disability for pneumoconiosis. Table E. The compensation standard of disability allowance for pneumoconiosis before retirement. Table F. The compensation standard of nursing cost for pneumoconiosis. Table G. Cumulative incidence density of CWP for tunneling workers. Table H. Logit variable transformation and incidence density estimation for tunneling workers. Table I. The number of CWP prediction for tunneling workers in the future if dust concentration maintained at the level of 2011. Table J. The number of CWP prediction for tunneling workers in the future if dust concentration maintained at the level of 2011. Table K. The number of CWP prediction for mining workers in the future if dust concentration maintained at the level of 2011. Table L. The number of CWP prediction for combining workers in the future if dust concentration maintained at the level of 2011. Table M. The number of CWP prediction for helping workers in the future if dust concentration maintained at the level of 2011. Table N. The number of CWP prediction for tunneling workers in the future if advanced dustproof measures was adopted. Table O. The number of CWP prediction for mining workers in the future if advanced dustproof measures was adopted. Table P. The number of CWP prediction for combining workers in the future if advanced dustproof measures was adopted. Table Q. The number of CWP prediction for helping workers in the future if advanced dustproof measures was adopted. Table R. Medical costs attributed to CWP. Table S. Lump-sum grants for disability caused by CWP. Table T. Allowances for disability caused by CWP. Table U. Nursing costs attributed to CWP. Table V. The age of onset di [file pone.0130958.s002.doc]

**Cost-effectiveness of coal workers' pneumoconiosis prevention based on its predicted incidence within the Datong Coal Mine Group in China**

S1 File. Supplementary tables A-AA**.**

Table A. The relationship between the disability grade of occupational disease and the stage of pneumoconiosis

| Disability grade of occupational disease | Stage of pneumoconiosis |
| --- | --- |
| Grade 1 | Stage Ⅲ of pneumoconiosis with severe injury of pulmonary function and/or severe hypoxemia |
| Grade 2 | Stage Ⅲ of pneumoconiosis with moderate injury of pulmonary function and/or moderate hypoxemia |
|  | Stage Ⅱ of pneumoconiosis with severe injury of pulmonary function and/or severe hypoxemia |
|  | Stage Ⅲ of pneumoconiosis with active tuberculosis |
| Grade 3 | Stage Ⅲ of pneumoconiosis |
|  | Stage Ⅱ of pneumoconiosis with moderate injury of pulmonary function and/or moderate hypoxemia |
|  | Stage Ⅱ of pneumoconiosis with active tuberculosis |
| Grade 4 | Stage Ⅱ of pneumoconiosis |
|  | Stage Ⅰ of pneumoconiosis with moderate injury of pulmonary function and/or moderate hypoxemia |
|  | Stage Ⅰ of pneumoconiosis with active tuberculosis |
| Grade 6 | Stage Ⅰ of pneumoconiosis with mild injury of pulmonary function and/or mild hypoxemia |
| Grade 7 | Stage Ⅰ of pneumoconiosis with normal lung function |

Table B. Cumulative incidence rate of CWP in the tunneling cohort

| Observed years | Initial observed person | Terminal observed person | Incidence number | Adjusted number | Incidence rate | No incidence rate | Cumulative no incidence rate | Cumulative incidence rate |
| --- | --- | --- | --- | --- | --- | --- | --- | --- |
| 0- | 3,369 | 34 | 0 | 3,352.0 | 0.0000 | 1.0000 | 1.0000 | 0.0000 |
| 2- | 3,335 | 140 | 0 | 3,265.0 | 0.0000 | 1.0000 | 1.0000 | 0.0000 |
| 4- | 3,195 | 41 | 0 | 3,174.5 | 0.0000 | 1.0000 | 1.0000 | 0.0000 |
| 6- | 3,154 | 23 | 12 | 3,142.5 | 0.0038 | 0.9962 | 0.9962 | 0.0038 |
| 8- | 3,119 | 30 | 3 | 3,104.0 | 0.0010 | 0.9990 | 0.9952 | 0.0048 |
| 10- | 3,086 | 39 | 46 | 3,066.5 | 0.0150 | 0.9850 | 0.9803 | 0.0197 |
| 12- | 3,001 | 308 | 62 | 2,847.0 | 0.0218 | 0.9782 | 0.9589 | 0.0411 |
| 14- | 2,631 | 53 | 100 | 2,604.5 | 0.0384 | 0.9616 | 0.9221 | 0.0779 |
| 16- | 2,478 | 369 | 87 | 2,293.5 | 0.0379 | 0.9621 | 0.8871 | 0.1129 |
| 18- | 2,022 | 77 | 52 | 1,983.5 | 0.0262 | 0.9738 | 0.8639 | 0.1361 |
| 20- | 1,893 | 80 | 48 | 1,853.0 | 0.0259 | 0.9741 | 0.8415 | 0.1585 |
| 22- | 1,765 | 80 | 51 | 1,725.0 | 0.0296 | 0.9704 | 0.8166 | 0.1834 |
| 24- | 1,634 | 58 | 29 | 1,605.0 | 0.0181 | 0.9819 | 0.8019 | 0.1981 |
| 26- | 1,547 | 357 | 45 | 1,368.5 | 0.0329 | 0.9671 | 0.7755 | 0.2245 |
| 28- | 1,145 | 198 | 37 | 1,046.0 | 0.0354 | 0.9646 | 0.7481 | 0.2519 |
| 30- | 910 | 106 | 28 | 857.0 | 0.0327 | 0.9673 | 0.7236 | 0.2764 |
| 32- | 776 | 119 | 32 | 716.5 | 0.0447 | 0.9553 | 0.6913 | 0.3087 |
| 34- | 625 | 74 | 35 | 588.0 | 0.0595 | 0.9405 | 0.6502 | 0.3498 |
| 36- | 516 | 192 | 30 | 420.0 | 0.0714 | 0.9286 | 0.6037 | 0.3963 |
| 38- | 294 | 106 | 6 | 241.0 | 0.0249 | 0.9751 | 0.5887 | 0.4113 |
| 40-42 | 182 | 150 | 32 | 107.0 | 0.2991 | 0.7009 | 0.4126 | 0.5874 |

Table C. Contents included in direct and indirect economic loss caused by CWP

| Item | Content |
| --- | --- |
| Direct economic loss | Medical cost |
|  | Lump-sum grant of disability |
|  | Disability allowance |
|  | Lump-sum grant of death |
|  | Funeral grant |
|  | Nursing cost |
|  | Food allowance |
|  | Traffic fee |
|  | Dependent relatives pension cost |
| Indirect economic loss | Social productivity loss caused by CWP patient |
|  | Cost of training new employees supplemented |
|  | Traffic fee of accompany members for CWP patients |
|  | Social productivity loss caused by accompany members |
|  | Loss of stopping or cutting production |

Table D. The compensation standard of lump-sum grant of disability for pneumoconiosis

| Disability grade | Compensation standard |
| --- | --- |
| Grade 2 | Person wage × 25 months |
| Grade 3 | Person wage × 23 months |
| Grade 4 | Person wage × 21 months |
| Grade 6 | Person wage × 16 months |
| Grade 7 | Person wage × 13 months |

Table E. The compensation standard of disability allowance for pneumoconiosis before retirement

| Disability grade | Compensation standard |
| --- | --- |
| Grade 2 | Person wage × 85% |
| Grade 3 | Person wage × 80% |
| Grade 4 | Person wage × 75% |
| Grade 6 | Person wage × 60% |

Table F. The compensation standard of nursing cost for pneumoconiosis

| Disability grade | Compensation standard |
| --- | --- |
| Grade 2 | Average wage of staff and workers by region × 40% |
| Grade 3 | Average wage of staff and workers by region × 30% |
| Grade 4 | Average wage of staff and workers by region × 30% |

Table G. Cumulative incidence density of CWP for tunneling workers

| Cumulative dust exposure (mg·years) | Initial observed person years | Terminal observed person years | Incidence person years | Incidence number of CWP | Adjusted person years | Incidence density | No incidence density | Cumulative no incidence density | Cumulative incidence density |
| --- | --- | --- | --- | --- | --- | --- | --- | --- | --- |
| 0- | 76,473.0 | 264.2 | 77.6 | 2 | 7,6340.9 | 0.000026 | 0.999974 | 0.999974 | 0.000026 |
| 200- | 76,131.2 | 272.7 | 0.0 | 0 | 75,994.9 | 0.000000 | 1.000000 | 0.999974 | 0.000026 |
| 400- | 75,858.5 | 245.8 | 0.0 | 0 | 75,735.6 | 0.000000 | 1.000000 | 0.999974 | 0.000026 |
| 600- | 75,612.8 | 240.0 | 86.5 | 12 | 75,492.7 | 0.000159 | 0.999841 | 0.999815 | 0.000185 |
| 800- | 75,286.2 | 129.4 | 0.0 | 0 | 75,221.5 | 0.000000 | 1.000000 | 0.999815 | 0.000185 |
| 1000- | 75,156.8 | 180.5 | 0.0 | 0 | 75,066.6 | 0.000000 | 1.000000 | 0.999815 | 0.000185 |
| 1200- | 74,976.4 | 251.6 | 10.5 | 1 | 74,850.6 | 0.000013 | 0.999987 | 0.999801 | 0.000199 |
| 1400- | 74,714.4 | 263.1 | 29.6 | 3 | 74,582.8 | 0.000040 | 0.999960 | 0.999761 | 0.000239 |
| 1600- | 74,421.6 | 2,110.7 | 11.0 | 1 | 73,366.3 | 0.000014 | 0.999986 | 0.999748 | 0.000252 |
| 1800- | 72,300.0 | 884.5 | 17.0 | 1 | 71,857.8 | 0.000014 | 0.999986 | 0.999734 | 0.000266 |
| 2000- | 71,398.5 | 449.0 | 74.3 | 5 | 71,174.0 | 0.000070 | 0.999930 | 0.999664 | 0.000336 |
| 2200- | 70,875.3 | 814.1 | 160.8 | 10 | 70,468.2 | 0.000142 | 0.999858 | 0.999522 | 0.000478 |
| 2400- | 69,900.4 | 1,060.8 | 376.8 | 24 | 69,370.0 | 0.000346 | 0.999654 | 0.999176 | 0.000824 |
| 2600- | 68,462.9 | 517.7 | 837.8 | 61 | 68,204.0 | 0.000894 | 0.999106 | 0.998282 | 0.001718 |
| 2800- | 67,107.5 | 782.8 | 730.3 | 45 | 66,716.0 | 0.000675 | 0.999325 | 0.997609 | 0.002391 |
| 3000- | 65,594.4 | 6,484.6 | 1072.2 | 67 | 62,352.1 | 0.001075 | 0.998925 | 0.996537 | 0.003463 |
| 3200- | 58,037.6 | 710.7 | 1053.2 | 61 | 57,682.2 | 0.001058 | 0.998942 | 0.995483 | 0.004517 |
| 3400- | 56,273.7 | 819.9 | 473.9 | 28 | 55,863.7 | 0.000501 | 0.999499 | 0.994984 | 0.005016 |
| 3600- | 54,979.9 | 1,744.9 | 701.9 | 40 | 54,107.5 | 0.000739 | 0.999261 | 0.994249 | 0.005751 |
| 3800- | 52,533.2 | 593.0 | 544.6 | 29 | 52,236.7 | 0.000555 | 0.999445 | 0.993697 | 0.006303 |
| 4000- | 51,395.6 | 1,751.7 | 617.0 | 31 | 50,519.8 | 0.000614 | 0.999386 | 0.993087 | 0.006913 |
| 4200- | 49,026.9 | 637.2 | 494.1 | 20 | 48,708.3 | 0.000411 | 0.999589 | 0.992679 | 0.007321 |
| 4400- | 47,895.6 | 1,031.1 | 710.3 | 30 | 47,380.1 | 0.000633 | 0.999367 | 0.992050 | 0.007950 |
| 4600- | 46,154.3 | 1,090.8 | 552.9 | 22 | 45,608.9 | 0.000482 | 0.999518 | 0.991572 | 0.008428 |
| 4800- | 44,510.6 | 1,068.1 | 238.9 | 10 | 43,976.6 | 0.000227 | 0.999773 | 0.991346 | 0.008654 |
| 5000- | 43,203.6 | 1,202.1 | 461.9 | 18 | 42,602.6 | 0.000423 | 0.999577 | 0.990928 | 0.009072 |
| 5200- | 41,539.6 | 7,733.5 | 710.7 | 26 | 37,672.8 | 0.000690 | 0.999310 | 0.990244 | 0.009756 |
| 5400- | 33,095.4 | 3,298.0 | 513.4 | 18 | 31,446.4 | 0.000572 | 0.999428 | 0.989677 | 0.010323 |
| 5600- | 29,284.0 | 3,896.7 | 819.0 | 30 | 27,335.6 | 0.001097 | 0.998903 | 0.988591 | 0.011409 |
| 5800- | 24,568.2 | 1,981.4 | 474.2 | 16 | 23,577.5 | 0.000679 | 0.999321 | 0.987920 | 0.012080 |
| 6000- | 22,112.6 | 1,538.5 | 245.0 | 8 | 21,343.4 | 0.000375 | 0.999625 | 0.987550 | 0.012450 |
| 6200- | 20,329.1 | 3,880.4 | 475.5 | 14 | 18,388.9 | 0.000761 | 0.999239 | 0.986798 | 0.013202 |
| 6400- | 15,973.2 | 527.0 | 305.4 | 9 | 15,709.7 | 0.000573 | 0.999427 | 0.986232 | 0.013768 |
| 6600- | 15,140.8 | 1,515.0 | 776.9 | 24 | 14,383.3 | 0.001669 | 0.998331 | 0.984587 | 0.015413 |
| 6800- | 12,848.9 | 1,348.3 | 694.2 | 21 | 12,174.8 | 0.001725 | 0.998275 | 0.982889 | 0.017111 |
| 7000- | 10,806.5 | 1,714.1 | 403.4 | 12 | 9,949.4 | 0.001206 | 0.998794 | 0.981703 | 0.018297 |
| 7200- | 8,689.0 | 1,766.6 | 391.1 | 11 | 7,805.7 | 0.001409 | 0.998591 | 0.980320 | 0.019680 |
| 7400- | 6,531.3 | 3,563.3 | 478.4 | 14 | 4,749.6 | 0.002948 | 0.997052 | 0.977430 | 0.022570 |
| 7600- | 2,489.6 | 1,577.5 | 417.3 | 10 | 1,700.8 | 0.005879 | 0.994121 | 0.971683 | 0.028317 |
| 7800- | 494.8 | 165.2 | 37.8 | 1 | 412.1 | 0.002426 | 0.997574 | 0.969326 | 0.030674 |
| 8000- | 291.7 | 291.7 | 0.0 | 0 | 145.9 | 0.000000 | 1.000000 | 0.969326 | 0.030674 |

Table H. Logit variable transformation and incidence density estimation for tunneling workers

| Cumulative dust exposure (mg·years) | Cumulative incidence density | Natural logarithm  of cumulative dust exposure up limit | logit | Logit estimation | Cumalative incidence density estimation | Cumalative no incidence density estimation | No incidence density estimation | Incidence density estimation |
| --- | --- | --- | --- | --- | --- | --- | --- | --- |
| 0- | 0.000026 | 5.30 | -10.55 | -14.62 | 0.000000 | 1.000000 | 1.000000 | 0.000000 |
| 200- | 0.000026 | 5.99 | -10.55 | -12.51 | 0.000004 | 0.999996 | 0.999997 | 0.000003 |
| 400- | 0.000026 | 6.40 | -10.55 | -11.28 | 0.000013 | 0.999987 | 0.999991 | 0.000009 |
| 600- | 0.000185 | 6.68 | -8.59 | -10.41 | 0.000030 | 0.999970 | 0.999982 | 0.000018 |
| 800- | 0.000185 | 6.91 | -8.59 | -9.73 | 0.000059 | 0.999941 | 0.999971 | 0.000029 |
| 1,000- | 0.000185 | 7.09 | -8.59 | -9.18 | 0.000103 | 0.999897 | 0.999956 | 0.000044 |
| 1,200- | 0.000199 | 7.24 | -8.52 | -8.71 | 0.000165 | 0.999835 | 0.999938 | 0.000062 |
| 1,400- | 0.000239 | 7.38 | -8.34 | -8.30 | 0.000248 | 0.999752 | 0.999917 | 0.000083 |
| 1,600- | 0.000252 | 7.50 | -8.28 | -7.95 | 0.000354 | 0.999646 | 0.999894 | 0.000106 |
| 1,800- | 0.000266 | 7.60 | -8.23 | -7.63 | 0.000488 | 0.999512 | 0.999867 | 0.000133 |
| 2,000- | 0.000336 | 7.70 | -8.00 | -7.34 | 0.000651 | 0.999349 | 0.999836 | 0.000164 |
| 2,200- | 0.000478 | 7.78 | -7.64 | -7.07 | 0.000848 | 0.999152 | 0.999803 | 0.000197 |
| 2,400- | 0.000824 | 7.86 | -7.10 | -6.83 | 0.001081 | 0.998919 | 0.999767 | 0.000233 |
| 2,600- | 0.001718 | 7.94 | -6.36 | -6.60 | 0.001353 | 0.998647 | 0.999727 | 0.000273 |
| 2,800- | 0.002391 | 8.01 | -6.03 | -6.39 | 0.001668 | 0.998332 | 0.999685 | 0.000315 |
| 3,000- | 0.003463 | 8.07 | -5.66 | -6.20 | 0.002028 | 0.997972 | 0.999639 | 0.000361 |
| 3,200- | 0.004517 | 8.13 | -5.40 | -6.01 | 0.002437 | 0.997563 | 0.999590 | 0.000410 |
| 3,400- | 0.005016 | 8.19 | -5.29 | -5.84 | 0.002898 | 0.997102 | 0.999538 | 0.000462 |
| 3,600- | 0.005751 | 8.24 | -5.15 | -5.68 | 0.003413 | 0.996587 | 0.999483 | 0.000517 |
| 3,800- | 0.006303 | 8.29 | -5.06 | -5.52 | 0.003986 | 0.996014 | 0.999425 | 0.000575 |
| 4,000- | 0.006913 | 8.34 | -4.97 | -5.37 | 0.004620 | 0.995380 | 0.999364 | 0.000636 |
| 4,200- | 0.007321 | 8.39 | -4.91 | -5.23 | 0.005317 | 0.994683 | 0.999300 | 0.000700 |
| 4,400- | 0.007950 | 8.43 | -4.83 | -5.10 | 0.006080 | 0.993920 | 0.999232 | 0.000768 |
| 4,600- | 0.008428 | 8.48 | -4.77 | -4.97 | 0.006913 | 0.993087 | 0.999162 | 0.000838 |
| 4,800- | 0.008654 | 8.52 | -4.74 | -4.84 | 0.007819 | 0.992181 | 0.999089 | 0.000911 |
| 5,000- | 0.009072 | 8.56 | -4.69 | -4.72 | 0.008799 | 0.991201 | 0.999012 | 0.000988 |
| 5,200- | 0.009756 | 8.59 | -4.62 | -4.61 | 0.009857 | 0.990143 | 0.998933 | 0.001067 |
| 5,400- | 0.010323 | 8.63 | -4.56 | -4.50 | 0.010995 | 0.989005 | 0.998851 | 0.001149 |
| 5,600- | 0.011409 | 8.67 | -4.46 | -4.39 | 0.012216 | 0.987784 | 0.998765 | 0.001235 |
| 5,800- | 0.012080 | 8.70 | -4.40 | -4.29 | 0.013522 | 0.986478 | 0.998677 | 0.001323 |
| 6,000- | 0.012450 | 8.73 | -4.37 | -4.19 | 0.014917 | 0.985083 | 0.998586 | 0.001414 |
| 6,200- | 0.013202 | 8.76 | -4.31 | -4.09 | 0.016402 | 0.983598 | 0.998493 | 0.001507 |
| 6,400- | 0.013768 | 8.79 | -4.27 | -4.00 | 0.017979 | 0.982021 | 0.998396 | 0.001604 |
| 6,600- | 0.015413 | 8.82 | -4.16 | -3.91 | 0.019652 | 0.980348 | 0.998297 | 0.001703 |
| 6,800- | 0.017111 | 8.85 | -4.05 | -3.82 | 0.021421 | 0.978579 | 0.998195 | 0.001805 |
| 7,000- | 0.018297 | 8.88 | -3.98 | -3.74 | 0.023289 | 0.976711 | 0.998091 | 0.001909 |
| 7,200- | 0.019680 | 8.91 | -3.91 | -3.65 | 0.025259 | 0.974741 | 0.997984 | 0.002016 |
| 7,400- | 0.022570 | 8.94 | -3.77 | -3.57 | 0.027331 | 0.972669 | 0.997874 | 0.002126 |
| 7,600- | 0.028317 | 8.96 | -3.54 | -3.49 | 0.029508 | 0.970492 | 0.997762 | 0.002238 |
| 7,800- | 0.030674 | 8.99 | -3.45 | -3.42 | 0.031791 | 0.968209 | 0.997648 | 0.002352 |
| 8,000- | 0.030674 | 9.01 | -3.45 | -3.34 | 0.034181 | 0.965819 | 0.997531 | 0.002469 |
| 8,200- | - | 9.04 | ­- | -3.27 | 0.036681 | 0.963319 | 0.997412 | 0.002588 |

Table I. The number of CWP prediction for tunneling workers in the future if dust concentration maintained at the level of 2011

| Cumulative dust exposure (mg·years) | Incidence density estimation | Initial observed person years | Terminal observed person years | Adjusted person years | Prediction incidence number |
| --- | --- | --- | --- | --- | --- |
| 0- | 0.000000 | 155,433.7 | 0.0 | 155,433.7 | 0.1 |
| 200- | 0.000003 | 155,433.7 | 0.0 | 155,433.7 | 0.5 |
| 400- | 0.000009 | 155,433.7 | 56.6 | 155,405.4 | 1.4 |
| 600- | 0.000018 | 155,377.2 | 114.8 | 155,319.7 | 2.7 |
| 800- | 0.000029 | 155,262.3 | 180.0 | 155,172.3 | 4.5 |
| 1,000- | 0.000044 | 155,082.3 | 5,400.2 | 152,382.2 | 6.7 |
| 1,200- | 0.000062 | 149,682.1 | 6,705.5 | 146,329.4 | 9.0 |
| 1,400- | 0.000083 | 142,976.7 | 1,434.8 | 142,259.3 | 11.7 |
| 1,600- | 0.000106 | 141,541.9 | 1,036.3 | 141,023.7 | 15.0 |
| 1,800- | 0.000133 | 140,505.6 | 1,287.2 | 139,862.0 | 18.7 |
| 2,000- | 0.000164 | 139,218.4 | 1,149.4 | 138,643.7 | 22.7 |
| 2,200- | 0.000197 | 138,069.0 | 983.3 | 137,577.3 | 27.1 |
| 2,400- | 0.000233 | 137,085.7 | 7,059.6 | 133,555.8 | 31.1 |
| 2,600- | 0.000273 | 130,026.0 | 7,021.8 | 126,515.1 | 34.5 |
| 2,800- | 0.000315 | 123,004.3 | 2,659.7 | 121,674.4 | 38.4 |
| 3,000- | 0.000361 | 120,344.6 | 4,281.6 | 118,203.8 | 42.7 |
| 3,200- | 0.000410 | 116,063.0 | 2,039.0 | 115,043.5 | 47.1 |
| 3,400- | 0.000462 | 114,024.0 | 6,039.7 | 111,004.2 | 51.3 |
| 3,600- | 0.000517 | 107,984.3 | 14,034.0 | 100,967.3 | 52.2 |
| 3,800- | 0.000575 | 93,950.3 | 7,044.3 | 90,428.2 | 52.0 |
| 4,000- | 0.000636 | 86,906.1 | 2,825.2 | 85,493.5 | 54.4 |
| 4,200- | 0.000700 | 84,080.9 | 3,267.8 | 82,447.0 | 57.7 |
| 4,400- | 0.000768 | 80,813.1 | 2,503.0 | 79,561.6 | 61.1 |
| 4,600- | 0.000838 | 78,310.1 | 2,389.1 | 77,115.6 | 64.6 |
| 4,800- | 0.000911 | 75,921.0 | 1,943.1 | 74,949.5 | 68.3 |
| 5,000- | 0.000988 | 73,977.9 | 2,623.6 | 72,666.2 | 71.8 |
| 5,200- | 0.001067 | 71,354.4 | 2,425.2 | 70,141.8 | 74.9 |
| 5,400- | 0.001149 | 68,929.2 | 9,493.3 | 64,182.6 | 73.8 |
| 5,600- | 0.001235 | 59,436.0 | 11,849.9 | 53,511.0 | 66.1 |
| 5,800- | 0.001323 | 47,586.0 | 7,131.9 | 44,020.1 | 58.2 |
| 6,000- | 0.001414 | 40,454.2 | 6,815.3 | 37,046.5 | 52.4 |
| 6,200- | 0.001507 | 33,638.9 | 4,415.4 | 31,431.2 | 47.4 |
| 6,400- | 0.001604 | 29,223.6 | 5,378.4 | 26,534.4 | 42.6 |
| 6,600- | 0.001703 | 23,845.2 | 4,027.9 | 21,831.2 | 37.2 |
| 6,800- | 0.001805 | 19,817.3 | 1,907.6 | 18,863.5 | 34.0 |
| 7,000- | 0.001909 | 17,909.7 | 3,738.9 | 16,040.3 | 30.6 |
| 7,200- | 0.002016 | 14,170.8 | 3,293.9 | 12,523.9 | 25.3 |
| 7,400- | 0.002126 | 10,877.0 | 4,301.3 | 8,726.3 | 18.6 |
| 7,600- | 0.002238 | 6,575.7 | 4,280.0 | 4,435.7 | 9.9 |
| 7,800- | 0.002352 | 2,295.7 | 1,841.3 | 1,375.0 | 3.2 |
| 8,000- | 0.002469 | 454.3 | 387.9 | 260.4 | 0.6 |
| 8,200- | 0.002588 | 66.4 | 66.4 | 33.2 | 0.1 |
| Total |  |  |  |  | 1,422.1 |

Table J. The number of CWP prediction for tunneling workers in the future if dust concentration maintained at the level of 2011

| Cumulative dust exposure (mg·years) | Adjusted person years | Incidence number of CWP | Cumulative incidence density | Natural logarithm  of cumulative dust exposure up limit | logit | Incidence density estimation | Adjusted person years for prediction | Prediction incidence number |
| --- | --- | --- | --- | --- | --- | --- | --- | --- |
| 0- | 76,340.9 | 2 | 0.000026 | 5.30 | -10.55 | 0.000000 | 155,433.7 | 0.1 |
| 200- | 75,994.9 | 0 | 0.000026 | 5.99 | -10.55 | 0.000003 | 155,433.7 | 0.5 |
| 400- | 75,735.6 | 0 | 0.000026 | 6.40 | -10.55 | 0.000009 | 155,405.4 | 1.4 |
| 600- | 75,492.7 | 12 | 0.000185 | 6.68 | -8.59 | 0.000018 | 155,319.7 | 2.7 |
| 800- | 75,221.5 | 0 | 0.000185 | 6.91 | -8.59 | 0.000029 | 155,172.3 | 4.5 |
| 1,000- | 75,066.6 | 0 | 0.000185 | 7.09 | -8.59 | 0.000044 | 152,382.2 | 6.7 |
| 1,200- | 74,850.6 | 1 | 0.000199 | 7.24 | -8.52 | 0.000062 | 146,329.4 | 9.0 |
| 1,400- | 74,582.8 | 3 | 0.000239 | 7.38 | -8.34 | 0.000083 | 142,259.3 | 11.7 |
| 1,600- | 73,366.3 | 1 | 0.000252 | 7.50 | -8.28 | 0.000106 | 141,023.7 | 15.0 |
| 1,800- | 71,857.8 | 1 | 0.000266 | 7.60 | -8.23 | 0.000133 | 139,862.0 | 18.7 |
| 2,000- | 71,174.0 | 5 | 0.000336 | 7.70 | -8.00 | 0.000164 | 138,643.7 | 22.7 |
| 2,200- | 70,468.2 | 10 | 0.000478 | 7.78 | -7.64 | 0.000197 | 137,577.3 | 27.1 |
| 2,400- | 69,370.0 | 24 | 0.000824 | 7.86 | -7.10 | 0.000233 | 133,555.8 | 31.1 |
| 2,600- | 68,204.0 | 61 | 0.001718 | 7.94 | -6.36 | 0.000273 | 126,515.1 | 34.5 |
| 2,800- | 66,716.0 | 45 | 0.002391 | 8.01 | -6.03 | 0.000315 | 121,674.4 | 38.4 |
| 3,000- | 62,352.1 | 67 | 0.003463 | 8.07 | -5.66 | 0.000361 | 118,203.8 | 42.7 |
| 3,200- | 57,682.2 | 61 | 0.004517 | 8.13 | -5.40 | 0.000410 | 115,043.5 | 47.1 |
| 3,400- | 55,863.7 | 28 | 0.005016 | 8.19 | -5.29 | 0.000462 | 111,004.2 | 51.3 |
| 3,600- | 54,107.5 | 40 | 0.005751 | 8.24 | -5.15 | 0.000517 | 100,967.3 | 52.2 |
| 3,800- | 52,236.7 | 29 | 0.006303 | 8.29 | -5.06 | 0.000575 | 90,428.2 | 52.0 |
| 4,000- | 50,519.8 | 31 | 0.006913 | 8.34 | -4.97 | 0.000636 | 85,493.5 | 54.4 |
| 4,200- | 48,708.3 | 20 | 0.007321 | 8.39 | -4.91 | 0.000700 | 82,447.0 | 57.7 |
| 4,400- | 47,380.1 | 30 | 0.007950 | 8.43 | -4.83 | 0.000768 | 79,561.6 | 61.1 |
| 4,600- | 45,608.9 | 22 | 0.008428 | 8.48 | -4.77 | 0.000838 | 77,115.6 | 64.6 |
| 4,800- | 43,976.6 | 10 | 0.008654 | 8.52 | -4.74 | 0.000911 | 74,949.5 | 68.3 |
| 5,000- | 42,602.6 | 18 | 0.009072 | 8.56 | -4.69 | 0.000988 | 72,666.2 | 71.8 |
| 5,200- | 37,672.8 | 26 | 0.009756 | 8.59 | -4.62 | 0.001067 | 70,141.8 | 74.9 |
| 5,400- | 31,446.4 | 18 | 0.010323 | 8.63 | -4.56 | 0.001149 | 64,182.6 | 73.8 |
| 5,600- | 27,335.6 | 30 | 0.011409 | 8.67 | -4.46 | 0.001235 | 53,511.0 | 66.1 |
| 5,800- | 23,577.5 | 16 | 0.012080 | 8.70 | -4.40 | 0.001323 | 44,020.1 | 58.2 |
| 6,000- | 21,343.4 | 8 | 0.012450 | 8.73 | -4.37 | 0.001414 | 37,046.5 | 52.4 |
| 6,200- | 18,388.9 | 14 | 0.013202 | 8.76 | -4.31 | 0.001507 | 31,431.2 | 47.4 |
| 6,400- | 15,709.7 | 9 | 0.013768 | 8.79 | -4.27 | 0.001604 | 26,534.4 | 42.6 |
| 6,600- | 14,383.3 | 24 | 0.015413 | 8.82 | -4.16 | 0.001703 | 21,831.2 | 37.2 |
| 6,800- | 12,174.8 | 21 | 0.017111 | 8.85 | -4.05 | 0.001805 | 18,863.5 | 34.0 |
| 7,000- | 9,949.4 | 12 | 0.018297 | 8.88 | -3.98 | 0.001909 | 16,040.3 | 30.6 |
| 7,200- | 7,805.7 | 11 | 0.019680 | 8.91 | -3.91 | 0.002016 | 12,523.9 | 25.3 |
| 7,400- | 4,749.6 | 14 | 0.022570 | 8.94 | -3.77 | 0.002126 | 8,726.3 | 18.6 |
| 7,600- | 1,700.8 | 10 | 0.028317 | 8.96 | -3.54 | 0.002238 | 4,435.7 | 9.9 |
| 7,800- | 412.1 | 1 | 0.030674 | 8.99 | -3.45 | 0.002352 | 1,375.0 | 3.2 |
| 8,000- | 145.9 | 0 | 0.030674 | 9.01 | -3.45 | 0.002469 | 260.4 | 0.6 |
| 8,200- | - | - |  | 9.04 | ­- | 0.002588 | 33.2 | 0.1 |
| Total |  |  |  |  |  |  |  | 1,422.1 |

Table K. The number of CWP prediction for mining workers in the future if dust concentration maintained at the level of 2011

| Cumulative dust exposure (mg·years) | Adjusted person years | Incidence number of CWP | Cumulative incidence density | Natural logarithm  of cumulative dust exposure up limit | logit | Incidence density estimation | Adjusted person years for prediction | Prediction incidence number |
| --- | --- | --- | --- | --- | --- | --- | --- | --- |
| 0- | 228,593.6 | 1 | 0.000004 | 5.30 | -12.34 | 0.000003 | 596,618.2 | 1.6 |
| 200- | 225,349.2 | 1 | 0.000009 | 5.99 | -11.64 | 0.000014 | 596,375.3 | 8.1 |
| 400- | 220,236.7 | 10 | 0.000054 | 6.40 | -9.82 | 0.000031 | 595,835.1 | 18.4 |
| 600- | 207,561.0 | 15 | 0.000126 | 6.68 | -8.98 | 0.000054 | 589,753.9 | 31.6 |
| 800- | 194,052.5 | 30 | 0.000281 | 6.91 | -8.18 | 0.000081 | 548,920.8 | 44.3 |
| 1,000- | 176,578.7 | 29 | 0.000445 | 7.09 | -7.72 | 0.000112 | 497,013.9 | 55.6 |
| 1,200- | 158,921.3 | 8 | 0.000496 | 7.24 | -7.61 | 0.000147 | 436,500.7 | 64.2 |
| 1,400- | 150,897.7 | 18 | 0.000615 | 7.38 | -7.39 | 0.000186 | 350,075.1 | 65.0 |
| 1,600- | 142,422.9 | 22 | 0.000769 | 7.50 | -7.17 | 0.000228 | 290,802.4 | 66.3 |
| 1,800- | 134,623.4 | 31 | 0.000999 | 7.60 | -6.91 | 0.000273 | 264,353.0 | 72.3 |
| 2,000- | 126,232.7 | 39 | 0.001308 | 7.70 | -6.64 | 0.000322 | 242,943.3 | 78.2 |
| 2,200- | 110,264.1 | 34 | 0.001616 | 7.78 | -6.43 | 0.000373 | 221,649.0 | 82.8 |
| 2,400- | 93,545.1 | 48 | 0.002128 | 7.86 | -6.15 | 0.000428 | 186,536.0 | 79.8 |
| 2,600- | 83,250.6 | 36 | 0.002560 | 7.94 | -5.97 | 0.000485 | 149,620.0 | 72.6 |
| 2,800- | 75,212.7 | 62 | 0.003382 | 8.01 | -5.69 | 0.000545 | 129,872.8 | 70.8 |
| 3,000- | 68,410.2 | 22 | 0.003702 | 8.07 | -5.60 | 0.000607 | 112,758.0 | 68.5 |
| 3,200- | 61,979.4 | 39 | 0.004329 | 8.13 | -5.44 | 0.000672 | 99,912.5 | 67.2 |
| 3,400- | 54,157.4 | 22 | 0.004734 | 8.19 | -5.35 | 0.000740 | 87,770.8 | 64.9 |
| 3,600- | 48,007.4 | 36 | 0.005480 | 8.24 | -5.20 | 0.000810 | 75,072.2 | 60.8 |
| 3,800- | 39,623.5 | 31 | 0.006258 | 8.29 | -5.07 | 0.000882 | 64,249.8 | 56.7 |
| 4,000- | 31,039.1 | 19 | 0.006866 | 8.34 | -4.97 | 0.000956 | 52,156.4 | 49.9 |
| 4,200- | 22,422.6 | 26 | 0.008018 | 8.39 | -4.82 | 0.001033 | 37,285.0 | 38.5 |
| 4,400- | 11,953.9 | 26 | 0.010176 | 8.43 | -4.58 | 0.001111 | 20,573.9 | 22.9 |
| 4,600- | 4,203.7 | 12 | 0.013001 | 8.48 | -4.33 | 0.001192 | 8,599.3 | 10.2 |
| 4,800- | 1,211.4 | 1 | 0.013816 | 8.52 | -4.27 | 0.001274 | 2,586.1 | 3.3 |
| 5,000- | 283.7 | 1 | 0.017292 | 8.56 | -4.04 | 0.001359 | 502.7 | 0.7 |
| Total | - | 619 | - | - | - | - | - | 1,254.9 |

Table L. The number of CWP prediction for combining workers in the future if dust concentration maintained at the level of 2011

| Cumulative dust exposure (mg·years) | Adjusted person years | Incidence number of CWP | Cumulative incidence density | Natural logarithm  of cumulative dust exposure up limit | logit | Incidence density estimation | Adjusted person years for prediction | Prediction incidence number |
| --- | --- | --- | --- | --- | --- | --- | --- | --- |
| 0- | 93,386.9 | 0 | 0.000000 | 5.30 | - | 0.000000 | 275,194.2 | 0.0 |
| 200- | 92,329.2 | 0 | 0.000000 | 5.99 | - | 0.000000 | 275,194.2 | 0.0 |
| 400- | 91,415.2 | 0 | 0.000000 | 6.40 | - | 0.000000 | 275,194.2 | 0.0 |
| 600- | 86,484.0 | 0 | 0.000000 | 6.68 | - | 0.000000 | 273,417.4 | 0.0 |
| 800- | 79,974.8 | 0 | 0.000000 | 6.91 | - | 0.000000 | 256,185.1 | 0.1 |
| 1,000- | 69,650.2 | 0 | 0.000000 | 7.09 | - | 0.000002 | 235,500.2 | 0.4 |
| 1,200- | 59,684.9 | 0 | 0.000000 | 7.24 | - | 0.000005 | 205,204.1 | 1.0 |
| 1,400- | 56,818.1 | 1 | 0.000018 | 7.38 | -10.95 | 0.000013 | 152,542.1 | 1.9 |
| 1,600- | 54,306.9 | 0 | 0.000018 | 7.50 | -10.95 | 0.000029 | 117,528.1 | 3.4 |
| 1,800- | 51,986.3 | 4 | 0.000095 | 7.60 | -9.27 | 0.000060 | 106,794.6 | 6.4 |
| 2,000- | 49,373.4 | 13 | 0.000358 | 7.70 | -7.94 | 0.000116 | 99,150.3 | 11.5 |
| 2,200- | 41,605.0 | 12 | 0.000646 | 7.78 | -7.34 | 0.000211 | 91,512.9 | 19.3 |
| 2,400- | 31,640.7 | 20 | 0.001278 | 7.86 | -6.66 | 0.000367 | 74,341.7 | 27.2 |
| 2,600- | 26,164.9 | 23 | 0.002156 | 7.94 | -6.14 | 0.000610 | 53,660.2 | 32.7 |
| 2,800- | 23,511.4 | 24 | 0.003174 | 8.01 | -5.75 | 0.000979 | 42,564.4 | 41.7 |
| 3,000- | 21,666.4 | 22 | 0.004187 | 8.07 | -5.47 | 0.001522 | 35,714.6 | 54.3 |
| 3,200- | 19,315.1 | 33 | 0.005888 | 8.13 | -5.13 | 0.002298 | 31,765.4 | 73.0 |
| 3,400- | 16,639.7 | 34 | 0.007919 | 8.19 | -4.83 | 0.003385 | 27,412.4 | 92.8 |
| 3,600- | 14,337.7 | 48 | 0.011240 | 8.24 | -4.48 | 0.004871 | 23,033.6 | 112.2 |
| 3,800- | 11,213.2 | 21 | 0.013092 | 8.29 | -4.32 | 0.006861 | 19,415.6 | 133.2 |
| 4,000- | 8,948.3 | 19 | 0.015188 | 8.34 | -4.17 | 0.009473 | 15,445.2 | 146.3 |
| 4,200- | 6,681.2 | 13 | 0.017104 | 8.39 | -4.05 | 0.012830 | 11,669.8 | 149.7 |
| 4,400- | 3,481.2 | 9 | 0.019645 | 8.43 | -3.91 | 0.017056 | 7,135.2 | 121.7 |
| 4,600- | 11,41.5 | 1 | 0.020504 | 8.48 | -3.87 | 0.022260 | 3,239.8 | 72.1 |
| 4,800- | 530.7 | 0 | 0.020504 | 8.52 | -3.87 | 0.028520 | 1,294.1 | 36.9 |
| 5,000- | 185.0 | 0 | 0.020504 | 8.56 | -3.87 | 0.035867 | 540.6 | 19.4 |
| 5,200- | 48.6 | 0 | 0.020504 | 8.59 | -3.87 | 0.044260 | 235.8 | 10.4 |
| 5,400- | 48.6 | 0 | 0.020504 | 8.63 | -3.87 | 0.053575 | 120.1 | 6.4 |
| 5,600- | 48.6 | 0 | 0.020504 | 8.67 | -3.87 | 0.063602 | 120.1 | 7.6 |
| 5,800- | 37.9 | 0 | 0.020504 | 8.70 | -3.87 | 0.074056 | 120.1 | 8.9 |
| 6,000- | 13.6 | 0 | 0.020504 | 8.73 | -3.87 | 0.084598 | 60.1 | 5.1 |
| Total |  | - | - | ­- | -­ | - | - | 1,195.8 |

Table M. The number of CWP prediction for helping workers in the future if dust concentration maintained at the level of 2011

| Cumulative dust exposure (mg·years) | Adjusted person years | Incidence number of CWP | Cumulative incidence density | Natural logarithm  of cumulative dust exposure up limit | logit | Incidence density estimation | Adjusted person years for prediction | Prediction incidence number |
| --- | --- | --- | --- | --- | --- | --- | --- | --- |
| 0- | 499,141.1 | 0 | 0.000000 | 1.61 | - | 0.000000 | 1,544,764.7 | 0.0 |
| 5- | 417,647.2 | 2 | 0.000005 | 2.30 | -12.2492 | 0.000000 | 1,347,849.0 | 0.3 |
| 10- | 359,657.8 | 2 | 0.000010 | 2.71 | -11.4786 | 0.000001 | 969,434.9 | 0.9 |
| 15- | 342,685.0 | 1 | 0.000013 | 3.00 | -11.2302 | 0.000002 | 745,998.7 | 1.8 |
| 20- | 327,314.2 | 2 | 0.000019 | 3.22 | -10.8514 | 0.000005 | 692,437.3 | 3.3 |
| 25- | 316,086.5 | 1 | 0.000023 | 3.40 | -10.7001 | 0.000009 | 658,172.6 | 5.6 |
| 30- | 306,732.2 | 4 | 0.000036 | 3.56 | -10.2436 | 0.000014 | 623,958.2 | 8.6 |
| 35- | 297,185.4 | 7 | 0.000059 | 3.69 | -9.73562 | 0.000021 | 596,951.9 | 12.3 |
| 40- | 287,965.3 | 7 | 0.000083 | 3.81 | -9.39127 | 0.000030 | 565,113.3 | 16.7 |
| 45- | 247,588.7 | 7 | 0.000112 | 3.91 | -9.09947 | 0.000041 | 471,111.5 | 19.1 |
| 50- | 195,109.0 | 9 | 0.000158 | 4.01 | -8.7538 | 0.000054 | 362,887.0 | 19.6 |
| 55- | 166,870.4 | 4 | 0.000182 | 4.09 | -8.61241 | 0.000070 | 316,053.8 | 22.2 |
| 60- | 147,631.7 | 7 | 0.000229 | 4.17 | -8.38065 | 0.000089 | 283,278.2 | 25.2 |
| 65- | 124,277.8 | 8 | 0.000294 | 4.25 | -8.13311 | 0.000111 | 247,984.7 | 27.5 |
| 70- | 104,340.5 | 27 | 0.000552 | 4.32 | -7.50094 | 0.000136 | 201,513.3 | 27.4 |
| 75- | 93,276.7 | 16 | 0.000724 | 4.38 | -7.23041 | 0.000165 | 162,118.2 | 26.7 |
| 80- | 82,275.1 | 16 | 0.000918 | 4.44 | -6.99237 | 0.000197 | 137,263.2 | 27.0 |
| 85- | 68,512.5 | 22 | 0.001239 | 4.50 | -6.69234 | 0.000233 | 110,556.5 | 25.7 |
| 90- | 50,350.4 | 15 | 0.001536 | 4.55 | -6.47679 | 0.000273 | 79,728.9 | 21.7 |
| 95- | 34,377.3 | 10 | 0.001827 | 4.61 | -6.30335 | 0.000317 | 53,395.7 | 16.9 |
| 100- | 22,440.7 | 18 | 0.002627 | 4.65 | -5.9391 | 0.000366 | 34,256.7 | 12.5 |
| 105- | 13,536.2 | 9 | 0.003291 | 4.70 | -5.71339 | 0.000419 | 20,563.6 | 8.6 |
| 110- | 7,487.7 | 2 | 0.003557 | 4.74 | -5.63532 | 0.000477 | 11,896.4 | 5.7 |
| 115- | 3,278.6 | 0 | 0.003557 | 4.79 | -5.63532 | 0.000540 | 5,646.8 | 3.0 |
| 120- | 2,578.1 | 0 | 0.003557 | 4.83 | -5.63532 | 0.000608 | 4,442.2 | 2.7 |
| 125- | 2,537.7 | 0 | 0.003557 | 4.87 | -5.63532 | 0.000681 | 4,378.0 | 3.0 |
| 130- | 2,502.0 | 0 | 0.003557 | 4.91 | -5.63532 | 0.000760 | 4,315.6 | 3.3 |
| 135- | 2,486.2 | 0 | 0.003557 | 4.94 | -5.63532 | 0.000844 | 4,253.3 | 3.6 |
| 140- | 2,417.2 | 0 | 0.003557 | 4.98 | -5.63532 | 0.000934 | 4,131.0 | 3.9 |
| 145- | 2,314.0 | 0 | 0.003557 | 5.01 | -5.63532 | 0.001030 | 3,945.6 | 4.1 |
| 150- | 2,279.8 | 0 | 0.003557 | 5.04 | -5.63532 | 0.001131 | 3,882.5 | 4.4 |
| 155- | 1,674.1 | 0 | 0.003557 | 5.08 | -5.63532 | 0.001239 | 2,845.2 | 3.5 |
| 160- | 1,050.1 | 0 | 0.003557 | 5.11 | -5.63532 | 0.001354 | 1,777.4 | 2.4 |
| 165- | 900.8 | 0 | 0.003557 | 5.14 | -5.63532 | 0.001474 | 1,526.0 | 2.2 |
| 170- | 696.8 | 0 | 0.003557 | 5.16 | -5.63532 | 0.001601 | 1,183.3 | 1.9 |
| 175- | 588.1 | 0 | 0.003557 | 5.19 | -5.63532 | 0.001734 | 1,001.4 | 1.7 |
| 180- | 479.7 | 0 | 0.003557 | 5.22 | -5.63532 | 0.001874 | 941.3 | 1.8 |
| 185- | 407.0 | 0 | 0.003557 | 5.25 | -5.63532 | 0.002021 | 817.5 | 1.7 |
| 190- | 370.1 | 0 | 0.003557 | 5.27 | -5.63532 | 0.002174 | 693.8 | 1.5 |
| 195- | 333.3 | 0 | 0.003557 | 5.30 | -5.63532 | 0.002335 | 630.3 | 1.5 |
| 200- | 166.6 | 0 | 0.003557 | 5.86 | -5.63532 | 0.161479 | 283.4 | 45.8 |
| Total |  |  |  |  |  |  |  | 427.3 |

Table N. The number of CWP prediction for tunneling workers in the future if advanced dustproof measures was adopted

| Cumulative dust exposure (mg·years) | Incidence density estimation | Initial observed person years | Terminal observed person years | Adjusted person years | Prediction incidence number |
| --- | --- | --- | --- | --- | --- |
| 0- | 0.000000 | 155,433.7 | 0.0 | 155,433.7 | 0.1 |
| 200- | 0.000003 | 155,433.7 | 7,877.0 | 151,495.2 | 0.5 |
| 400- | 0.000009 | 147,556.8 | 3,788.8 | 145,662.3 | 1.3 |
| 600- | 0.000018 | 143,767.9 | 1,706.7 | 142,914.6 | 2.5 |
| 800- | 0.000029 | 142,061.3 | 908.3 | 141,607.1 | 4.1 |
| 1,000- | 0.000044 | 141,153.0 | 1,218.2 | 140,543.9 | 6.2 |
| 1,200- | 0.000062 | 139,934.8 | 702.2 | 139,583.7 | 8.6 |
| 1,400- | 0.000083 | 139,232.6 | 914.1 | 138,775.5 | 11.5 |
| 1,600- | 0.000106 | 138,318.5 | 637.0 | 138,000.0 | 14.7 |
| 1,800- | 0.000133 | 137,681.5 | 10,819.4 | 132,271.8 | 17.7 |
| 2,000- | 0.000164 | 126,862.1 | 4,266.7 | 124,728.8 | 20.4 |
| 2,200- | 0.000197 | 122,595.4 | 1,413.2 | 121,888.9 | 24.0 |
| 2,400- | 0.000233 | 121,182.3 | 3,771.5 | 119,296.5 | 27.8 |
| 2,600- | 0.000273 | 117,410.8 | 2,376.2 | 116,222.7 | 31.7 |
| 2,800- | 0.000315 | 115,034.6 | 1,694.3 | 114,187.4 | 36.0 |
| 3,000- | 0.000361 | 113,340.2 | 13,936.8 | 106,371.8 | 38.4 |
| 3,200- | 0.000410 | 99,403.4 | 9,397.4 | 94,704.8 | 38.8 |
| 3,400- | 0.000462 | 90,006.1 | 2,654.9 | 88,678.7 | 40.9 |
| 3,600- | 0.000517 | 87,351.2 | 4,168.7 | 85,266.9 | 44.1 |
| 3,800- | 0.000575 | 83,182.6 | 1,383.2 | 82,490.9 | 47.4 |
| 4,000- | 0.000636 | 81,799.3 | 2,622.4 | 80,488.2 | 51.2 |
| 4,200- | 0.000700 | 79,177.0 | 2,173.0 | 78,090.5 | 54.7 |
| 4,400- | 0.000768 | 77,003.9 | 1,291.1 | 76,358.4 | 58.6 |
| 4,600- | 0.000838 | 75,712.9 | 2,266.3 | 74,579.7 | 62.5 |
| 4,800- | 0.000911 | 73,446.6 | 2,402.1 | 72,245.6 | 65.9 |
| 5,000- | 0.000988 | 71,044.5 | 3431.2 | 69,328.9 | 68.5 |
| 5,200- | 0.001067 | 67,613.4 | 16,849.6 | 59,188.5 | 63.2 |
| 5,400- | 0.001149 | 50,763.7 | 6,012.8 | 47,757.3 | 54.9 |
| 5,600- | 0.001235 | 44,751.0 | 8,597.9 | 40,452.0 | 49.9 |
| 5,800- | 0.001323 | 36,153.1 | 4,177.4 | 34,064.4 | 45.1 |
| 6,000- | 0.001414 | 31,975.7 | 2,710.6 | 30,620.4 | 43.3 |
| 6,200- | 0.001507 | 29,265.1 | 4,873.9 | 26,828.2 | 40.4 |
| 6,400- | 0.001604 | 24,391.3 | 3,951.6 | 22,415.5 | 35.9 |
| 6,600- | 0.001703 | 20,439.7 | 2,401.2 | 19,239.1 | 32.8 |
| 6,800- | 0.001805 | 18,038.5 | 2,557.4 | 16,759.8 | 30.2 |
| 7,000- | 0.001909 | 15,481.0 | 3,069.8 | 13,946.1 | 26.6 |
| 7,200- | 0.002016 | 12,411.2 | 3,112.0 | 10,855.2 | 21.9 |
| 7,400- | 0.002126 | 9,299.2 | 4,721.1 | 6,938.7 | 14.8 |
| 7,600- | 0.002238 | 4,578.1 | 3,616.2 | 2,770.0 | 6.2 |
| 7,800- | 0.002352 | 962.0 | 507.6 | 708.1 | 1.7 |
| 8,000- | 0.002469 | 454.3 | 454.3 | 227.2 | 0.6 |
| Total |  |  |  |  | 1,245.5 |

Table O. The number of CWP prediction for mining workers in the future if advanced dustproof measures was adopted

| Cumulative dust exposure (mg·years) | Incidence density estimation | Initial observed person years | Terminal observed person years | Adjusted person years | Prediction incidence number |
| --- | --- | --- | --- | --- | --- |
| 0- | 0.000003 | 596,649.5 | 1,282.1 | 596,008.5 | 1.5 |
| 200- | 0.000014 | 595,367.5 | 65,594.8 | 562,570.0 | 7.6 |
| 400- | 0.000031 | 529,772.6 | 24,746.1 | 517,399.6 | 16.0 |
| 600- | 0.000054 | 505,026.5 | 33,313.6 | 488,369.7 | 26.1 |
| 800- | 0.000081 | 471,712.9 | 78,220.2 | 432,602.8 | 34.9 |
| 1,000- | 0.000112 | 393,492.7 | 74,082.7 | 356,451.3 | 39.9 |
| 1,200- | 0.000147 | 319,410.0 | 31,208.4 | 303,805.8 | 44.7 |
| 1,400- | 0.000186 | 288,201.6 | 20,197.8 | 278,102.7 | 51.7 |
| 1,600- | 0.000228 | 268,003.8 | 25,922.0 | 255,042.8 | 58.1 |
| 1,800- | 0.000273 | 242,081.8 | 14,965.8 | 234,598.9 | 64.1 |
| 2,000- | 0.000322 | 227,116.0 | 16,523.5 | 218,854.3 | 70.4 |
| 2,200- | 0.000373 | 210,592.5 | 49,156.1 | 186,014.5 | 69.5 |
| 2,400- | 0.000428 | 161,436.4 | 23,384.3 | 149,744.3 | 64.1 |
| 2,600- | 0.000485 | 138,052.2 | 13,140.9 | 131,481.7 | 63.8 |
| 2,800- | 0.000545 | 124,911.3 | 17,648.5 | 116,087.1 | 63.3 |
| 3,000- | 0.000607 | 107,262.8 | 6,605.4 | 103,960.1 | 63.1 |
| 3,200- | 0.000672 | 100,657.4 | 15,142.6 | 93,086.1 | 62.6 |
| 3,400- | 0.000740 | 85,514.8 | 8,958.1 | 81,035.7 | 60.0 |
| 3,600- | 0.000810 | 76,556.7 | 8,754.7 | 72,179.4 | 58.4 |
| 3,800- | 0.000882 | 67,802.0 | 15,699.7 | 59,952.2 | 52.9 |
| 4,000- | 0.000956 | 52,102.3 | 8,338.3 | 47,933.2 | 45.8 |
| 4,200- | 0.001033 | 43,764.1 | 17,622.0 | 34,953.0 | 36.1 |
| 4,400- | 0.001111 | 26,142.0 | 17,034.0 | 17,625.0 | 19.6 |
| 4,600- | 0.001192 | 9,108.1 | 5,839.0 | 6,188.5 | 7.4 |
| 4,800- | 0.001274 | 3,269.0 | 2,457.8 | 2,040.1 | 2.6 |
| 5,000- | 0.001359 | 811.2 | 811.2 | 405.6 | 0.6 |
| Total | - |  |  |  | 1,084.7 |

Table P. The number of CWP prediction for combining workers in the future if advanced dustproof measures was adopted

| Cumulative dust exposure (mg·years) | Incidence density estimation | Initial observed person years | Terminal observed person years | Adjusted person years | Prediction incidence number |
| --- | --- | --- | --- | --- | --- |
| 0- | 0.000000 | 275,194.2 | 108.1 | 275,140.1 | 0.0 |
| 200- | 0.000000 | 275,086.1 | 33,064.7 | 258,553.8 | 0.0 |
| 400- | 0.000000 | 242,021.4 | 7,489.8 | 238,276.6 | 0.0 |
| 600- | 0.000000 | 234,531.7 | 6,155.2 | 231,454.1 | 0.0 |
| 800- | 0.000000 | 228,376.5 | 48,784.2 | 203,984.4 | 0.1 |
| 1,000- | 0.000002 | 179,592.3 | 48,552.2 | 155,316.2 | 0.3 |
| 1,200- | 0.000005 | 131,040.0 | 17,227.4 | 122,426.4 | 0.6 |
| 1,400- | 0.000013 | 113,812.7 | 6,818.4 | 110,403.5 | 1.4 |
| 1,600- | 0.000029 | 106,994.3 | 7,456.0 | 103,266.3 | 3.0 |
| 1,800- | 0.000060 | 99,538.3 | 6,356.4 | 96,360.1 | 5.7 |
| 2,000- | 0.000116 | 93,181.9 | 4,798.9 | 90,782.4 | 10.5 |
| 2,200- | 0.000211 | 88,382.9 | 29,143.5 | 73,811.2 | 15.6 |
| 2,400- | 0.000367 | 59,239.4 | 13,107.1 | 52,685.9 | 19.3 |
| 2,600- | 0.000610 | 46,132.3 | 6,124.8 | 43,069.9 | 26.3 |
| 2,800- | 0.000979 | 40,007.5 | 6,880.3 | 36,567.4 | 35.8 |
| 3,000- | 0.001522 | 33,127.2 | 2,160.2 | 32,047.1 | 48.8 |
| 3,200- | 0.002298 | 30,967.1 | 5,343.2 | 28,295.5 | 65.0 |
| 3,400- | 0.003385 | 25,623.9 | 2,303.9 | 24,471.9 | 82.8 |
| 3,600- | 0.004871 | 23,320.0 | 3,786.0 | 21,427.0 | 104.4 |
| 3,800- | 0.006861 | 19,534.0 | 4,406.2 | 17,330.9 | 118.9 |
| 4,000- | 0.009473 | 15,127.8 | 2,351.7 | 13,952.0 | 132.2 |
| 4,200- | 0.012830 | 12,776.2 | 3,693.0 | 10,929.7 | 140.2 |
| 4,400- | 0.017056 | 9,083.2 | 6,403.2 | 5,881.5 | 100.3 |
| 4,600- | 0.022260 | 2,679.9 | 1,329.8 | 2,015.0 | 44.9 |
| 4,800- | 0.028520 | 1,350.2 | 751.4 | 974.5 | 27.8 |
| 5,000- | 0.035867 | 598.8 | 478.7 | 359.5 | 12.9 |
| 5,200- | 0.044260 | 120.1 | 0.0 | 120.1 | 5.3 |
| 5,400- | 0.053575 | 120.1 | 0.0 | 120.1 | 6.4 |
| 5,600- | 0.063602 | 120.1 | 0.0 | 120.1 | 7.6 |
| 5,800- | 0.074056 | 120.1 | 120.1 | 60.0 | 4.4 |
| Total | - |  |  | - | 1,020.5 |

Table Q. The number of CWP prediction for helping workers in the future if advanced dustproof measures was adopted

| Cumulative dust exposure (mg·years) | Incidence density estimation | Initial observed person years | Terminal observed person years | Adjusted person years | Prediction incidence number |
| --- | --- | --- | --- | --- | --- |
| 0- | 0.000000 | 1,544,764.7 | 359,406.6 | 1,365,061.4 | 0.0 |
| 5- | 0.000000 | 1,185,358.1 | 278,116.2 | 1,046,300.0 | 0.2 |
| 10- | 0.000001 | 907,241.9 | 153,879.4 | 830,302.2 | 0.8 |
| 15- | 0.000002 | 753,362.5 | 70,478.4 | 718,123.3 | 1.7 |
| 20- | 0.000005 | 682,884.1 | 45,028.0 | 660,370.1 | 3.2 |
| 25- | 0.000009 | 637,856.1 | 16,173.6 | 629,769.3 | 5.4 |
| 30- | 0.000014 | 621,682.5 | 32,377.4 | 605,493.8 | 8.3 |
| 35- | 0.000021 | 589,305.2 | 15,813.3 | 581,398.5 | 12.0 |
| 40- | 0.000030 | 573,491.8 | 27,594.2 | 559,694.7 | 16.5 |
| 45- | 0.000041 | 545,897.6 | 152,625.6 | 469,584.8 | 19.1 |
| 50- | 0.000054 | 393,272.0 | 61,389.4 | 362,577.3 | 19.6 |
| 55- | 0.000070 | 331,882.7 | 39,792.6 | 311,986.4 | 21.9 |
| 60- | 0.000089 | 292,090.1 | 49,249.2 | 267,465.5 | 23.8 |
| 65- | 0.000111 | 242,840.9 | 56,935.7 | 214,373.0 | 23.8 |
| 70- | 0.000136 | 185,905.2 | 25,591.2 | 173,109.6 | 23.5 |
| 75- | 0.000165 | 160,314.0 | 17,121.5 | 151,753.3 | 25.0 |
| 80- | 0.000197 | 143,192.5 | 20,784.5 | 132,800.2 | 26.1 |
| 85- | 0.000233 | 122,408.0 | 25,660.9 | 109,577.6 | 25.5 |
| 90- | 0.000273 | 96,747.1 | 34,477.3 | 79,508.5 | 21.7 |
| 95- | 0.000317 | 62,269.8 | 18,204.5 | 53,167.6 | 16.9 |
| 100- | 0.000366 | 44,065.3 | 20,070.6 | 34,030.0 | 12.4 |
| 105- | 0.000419 | 23,994.8 | 7,051.5 | 20,469.0 | 8.6 |
| 110- | 0.000477 | 16,943.3 | 10,348.4 | 11,769.1 | 5.6 |
| 115- | 0.000540 | 6,594.9 | 2,151.6 | 5,519.1 | 3.0 |
| 120- | 0.000608 | 4,443.3 | 65.3 | 4,410.6 | 2.7 |
| 125- | 0.000681 | 4,378.0 | 64.2 | 4,345.9 | 3.0 |
| 130- | 0.000760 | 4,313.8 | 60.6 | 4,283.6 | 3.3 |
| 135- | 0.000844 | 4,253.3 | 0.0 | 4,253.3 | 3.6 |
| 140- | 0.000934 | 4,253.3 | 244.6 | 4,131.0 | 3.9 |
| 145- | 0.001030 | 4,008.7 | 126.2 | 3,945.6 | 4.1 |
| 150- | 0.001131 | 3,882.5 | 0.0 | 3,882.5 | 4.4 |
| 155- | 0.001239 | 3,882.5 | 2,074.6 | 2,845.2 | 3.5 |
| 160- | 0.001354 | 1,807.9 | 61.0 | 1,777.4 | 2.4 |
| 165- | 0.001474 | 1,746.9 | 441.7 | 1,526.0 | 2.2 |
| 170- | 0.001601 | 1,305.2 | 243.7 | 1,183.3 | 1.9 |
| 175- | 0.001734 | 1,061.5 | 120.2 | 1,001.4 | 1.7 |
| 180- | 0.001874 | 941.3 | 247.5 | 817.5 | 1.5 |
| 185- | 0.002021 | 693.8 | 0.0 | 693.8 | 1.4 |
| 190- | 0.002174 | 693.8 | 127.0 | 630.3 | 1.4 |
| 195- | 0.002335 | 566.8 | 0.0 | 566.8 | 1.3 |
| 200- | 0.161479 | 566.8 | 566.8 | 283.4 | 45.8 |
| Total |  |  |  |  | 412.5 |

Table R. Medical costs attributed to CWP

| Age at CWP diagnosis (years) | n | Life expectancy (years) | Average duration of disability (years) | Average yearly medical costs (RMB) | Total medical costs (RMB) |
| --- | --- | --- | --- | --- | --- |
| 20- | 1 | 57.2 | 35.9 | 23,137.5 | 830,636.3 |
| 25- | 9 | 52.4 | 47.3 | 23,137.5 | 9,839,221.9 |
| 30- | 106 | 47.5 | 42.8 | 23,137.5 | 105,019,261.5 |
| 35- | 328 | 42.7 | 37.8 | 23,137.5 | 286,867,980.0 |
| 40- | 466 | 38.0 | 33.2 | 23,137.5 | 357,641,427.8 |
| 45- | 384 | 33.5 | 31.3 | 23,137.5 | 278,183,088.0 |
| 50- | 275 | 28.9 | 27.7 | 23,137.5 | 176,504,418.8 |
| 55- | 161 | 24.4 | 22.6 | 23,137.5 | 84,337,113.0 |
| 60- | 82 | 20.3 | 19.8 | 23,137.5 | 37,585,017.8 |
| 65- | 31 | 16.5 | 15.5 | 23,137.5 | 11,096,050.9 |
| 70- | 3 | 13.0 | 13.0 | 23,137.5 | 902,362.5 |
| 75- | 1 | 10.0 | 10.0 | 23,137.5 | 231,375.0 |
| Total | 1,847 | - | 31.6 | - | 1,349,037,953.3 |

Table S. Lump-sum grants for disability caused by CWP

| Disability grade | n | Average monthly wage (RMB) | Compensation standard (months) | Total compensation (RMB) |
| --- | --- | --- | --- | --- |
| Second | 8 | 8,056.2 | 25 | 1,611,230.0 |
| Third | 147 | 8,056.2 | 23 | 27,237,843.2 |
| Fourth | 500 | 8,056.2 | 21 | 84,589,575.0 |
| Sixth | 432 | 8,056.2 | 16 | 55,684,108.8 |
| Seventh | 760 | 8,056.2 | 13 | 79,594,762.0 |
| Total | 1,847 | - | - | 248,717,519.0 |

Table T. Allowances for disability caused by CWP

| Disability grade | n | Average age at CWP diagnosis (years) | Average length of compensation(months) | Average monthly wage (RMB) | Compensation standard (percent of wage) | Total compensation (RMB) |
| --- | --- | --- | --- | --- | --- | --- |
| Second | 8 | 44.5 | 185.8 | 8,056.2 | 0.85 | 10,176,818.7 |
| Third | 128 | 45.5 | 163.7 | 8,056.2 | 0.80 | 135,003,028.2 |
| Fourth | 479 | 42.5 | 201.2 | 8,056.2 | 0.75 | 582,191,616.9 |
| Sixth | 432 | 40.5 | 226.0 | 8,056.2 | 0.60 | 471,985,466.7 |
| Total | 1,047 | - | - | 8,056.2 | - | 1,199,356,930.5 |

Table U. Nursing costs attributed to CWP

| Disability grade | n | Average age at CWP diagnosis (years) | Average length of compensation(years) | Average yearly wage in Shanxi Province (RMB) | Compensation standard (percent of wage) | Total compensation (RMB) |
| --- | --- | --- | --- | --- | --- | --- |
| Second | 8 | 44.5 | 33.5 | 39,230 | 0.4 | 4,205,456.0 |
| Third | 147 | 47.9 | 30.4 | 39,230 | 0.3 | 52,506,805.1 |
| Fourth | 500 | 43.4 | 34.1 | 39,230 | 0.3 | 200,602,605.0 |
| Total | 655 |  |  | - | - | 257,314,866.1 |

Table V. The age of onset distribution of CWP in each occupational character

| Characteristics |  | n | Mean±S | Minimum | Maximum |
| --- | --- | --- | --- | --- | --- |
| Survival or death | Survival | 1,637 | 46.3±8.3 | 28.0 | 75.9 |
|  | Death | 210 | 42.9±7.0 | 22.0 | 68.8 |
| Stage of CWP | Ⅰ | 1,503 | 45.7±8.1 | 22.0 | 75.9 |
|  | Ⅱ | 215 | 46.0±8.5 | 30.0 | 71.8 |
|  | Ⅲ | 129 | 49.3±9.4 | 28.0 | 69.8 |
| Disability grade | Second | 8 | 44.5±4.1 | 39.0 | 51.5 |
|  | Third | 147 | 47.9±8.8 | 31.0 | 69.3 |
|  | Fourth | 500 | 43.5±7.8 | 22.0 | 71.8 |
|  | Sixth | 432 | 40.5±5.1 | 29.0 | 54.0 |
|  | Seventh | 760 | 50.3±7.5 | 29.0 | 75.9 |
| Total |  | 1,847 | 45.9±8.3 | 22.0 | 75.9 |

Table W. Years lived with disability (YLD) by CWP patients

| Age at CWP diagnosis (years) | n | Life expectancy (years) | Average length of disability (years) | YLD | Average YLD |
| --- | --- | --- | --- | --- | --- |
| 20- | 1 | 57.2 | 35.9 | 16.1 | 16.1 |
| 25- | 9 | 52.4 | 47.3 | 109.2 | 12.1 |
| 30- | 106 | 47.5 | 42.8 | 1,198.4 | 11.3 |
| 35- | 328 | 42.7 | 37.8 | 3,167.4 | 9.7 |
| 40- | 466 | 38.0 | 33.2 | 3,805.3 | 8.2 |
| 45- | 384 | 33.5 | 31.3 | 2,616.0 | 6.8 |
| 50- | 275 | 28.9 | 27.7 | 1,402.5 | 5.1 |
| 55- | 161 | 24.4 | 22.6 | 609.1 | 3.8 |
| 60- | 82 | 20.3 | 19.8 | 292.5 | 3.6 |
| 65- | 31 | 16.5 | 15.5 | 89.3 | 2.9 |
| 70- | 3 | 13.0 | 13.0 | 6.4 | 2.1 |
| 75- | 1 | 10.0 | 10.0 | 1.1 | 1.1 |
| Total | 1,847 | 34.8 | 31.6 | 13,313.4 | 7.2 |

Table X. Years of life lost (YLL) by CWP patients

| Age at CWP diagnosis (years) | n | Life expectancy (years) | Average age at death (years) | YLL | Average YLL |
| --- | --- | --- | --- | --- | --- |
| 20- | 1 | 57.2 | 57.9 | 11.7 | 11.7 |
| 25- | 1 | 52.4 | 35.1 | 26.3 | 26.0 |
| 30- | 15 | 47.5 | 47.7 | 273.6 | 18.2 |
| 35- | 56 | 42.7 | 51.4 | 887.0 | 15.8 |
| 40- | 65 | 38.0 | 56.3 | 839.4 | 12.9 |
| 45- | 42 | 33.5 | 60.3 | 453.1 | 10.8 |
| 50- | 14 | 28.9 | 57.6 | 171.8 | 12.3 |
| 55- | 12 | 24.4 | 57.4 | 150.4 | 12.5 |
| 60- | 2 | 20.3 | 62.4 | 20.0 | 10.0 |
| 65- | 2 | 16.5 | 57.6 | 15.5 | 7.8 |
| Total | 210 | - | 54.4 | 2,848.8 | 13.6 |

Table Y. Disability adjusted life years (DALY) caused by CWP (year)

| DALY | n | Total healthy life loss | Mean |
| --- | --- | --- | --- |
| YLD | 1,847 | 13,313.4 | 7.2 |
| YLL | 210 | 2,848.8 | 13.6 |
| DALY | 1847 | 16,162.2 | 8.8 |

Table Z. Economic losses attributed to decreased social productivity by patients with CWP

| Age at CWP diagnosis (years) | n | YLD | YLL | DALY | Weight of productivity | Per capita GDP (RMB) | Loss of social productivity (RMB) |
| --- | --- | --- | --- | --- | --- | --- | --- |
| 20- | 1 | 16.1 | 11.7 | 27.8 | 0.75 | 35,181 | 733,787.7 |
| 25- | 9 | 109.2 | 26.3 | 135.5 | 0.75 | 35,181 | 3,574,213.7 |
| 30- | 106 | 1,198.4 | 273.6 | 1,472.0 | 0.75 | 35,181 | 38,840,351.7 |
| 35- | 328 | 3,167.4 | 887.0 | 4,054.4 | 0.75 | 35,181 | 106,977,065.5 |
| 40- | 466 | 3,805.3 | 839.4 | 4,644.7 | 0.75 | 35,181 | 122,554,156.9 |
| 45- | 384 | 2,616.0 | 453.1 | 3,069.1 | 0.80 | 35,181 | 86,380,331.5 |
| 50- | 275 | 1,402.5 | 171.8 | 1,574.3 | 0.80 | 35,181 | 44,308,358.6 |
| 55- | 161 | 609.1 | 150.4 | 759.5 | 0.80 | 35,181 | 21,377,101.4 |
| 60- | 82 | 292.5 | 20.0 | 312.5 | 0.10 | 35,181 | 1,099,335.9 |
| 65- | 31 | 89.3 | 15.5 | 104.8 | 0.10 | 35,181 | 368,802.4 |
| 70- | 3 | 6.4 | 0 | 6.4 | 0.10 | 35,181 | 22,445.5 |
| 75- | 1 | 1.1 | 0 | 1.1 | 0.10 | 35,181 | 3,975.5 |
| Total | 1,847 | 13,313.4 | 2,848.8 | 16,162.2 | - | - | 426,239,926.3 |

DALY: disability-adjusted life years; GDP: gross domestic product; YLD: years lived with disability; YLL: years of life lost.

Table AA. Total economic loss caused by CWP (RMB)

| Item | Economic loss | Proportion (%) |
| --- | --- | --- |
| Direct economic loss | 3,488,134,597.0 | 84.0 |
| Indirect economic loss | 663,187,570.9 | 16.0 |
| Total | 4,151,322,167.9 | 100.0 |
